# Antigen identification strategies and preclinical evaluation models for advancing tuberculosis vaccine development

**DOI:** 10.1038/s41541-024-00834-y

**Published:** 2024-03-09

**Authors:** Saurabh Chugh, Ritika Kar Bahal, Rohan Dhiman, Ramandeep Singh

**Affiliations:** 1 Centre for Tuberculosis Research, Tuberculosis Research Laboratory, Translational Health Science and Technology Institute, Faridabad, 121001 Haryana India; 2 Marshall Centre, School of Biomedical Sciences, University of Western Australia, Perth, Australia; 3 Laboratory of Mycobacterial Immunology, Department of Life Science, National Institute of Technology, Rourkela, 769008 Odisha India

**Keywords:** Bacterial infection, Bacterial infection

## Abstract

In its myriad devastating forms, Tuberculosis (TB) has existed for centuries, and humanity is still affected by it. *Mycobacterium tuberculosis* (*M. tuberculosis*), the causative agent of TB, was the foremost killer among infectious agents until the COVID-19 pandemic. One of the key healthcare strategies available to reduce the risk of TB is immunization with bacilli Calmette-Guerin (BCG). Although BCG has been widely used to protect against TB, reports show that BCG confers highly variable efficacy (0-80%) against adult pulmonary TB. Unwavering efforts have been made over the past 20 years to develop and evaluate new TB vaccine candidates. The failure of conventional preclinical animal models to fully recapitulate human response to TB, as also seen for the failure of MVA85A in clinical trials, signifies the need to develop better preclinical models for TB vaccine evaluation. In the present review article, we outline various approaches used to identify protective mycobacterial antigens and recent advancements in preclinical models for assessing the efficacy of candidate TB vaccines.

## Introduction

Tuberculosis (TB) remains a major global health concern that accounts for nearly 4000 lives daily. Bacilli Calmette-Guerin (BCG), the only licensed and century-old vaccine, has conferred variable protection in several field trials^1–4^. Developing better vaccines has been a crucial objective in TB research. The decoding of the complete genome of *M. tuberculosis* in 1998 represents a breakthrough in TB vaccine research and has resulted in significant advances in the area of TB vaccine antigen discovery^5^. Several different approaches have been used to identify antigens for developing TB vaccines over the past two decades. Identifying *M. tuberculosis* antigens and epitopes has enhanced our knowledge of the *M. tuberculosis* antigenome, leading to the identification of various candidates for TB vaccine development. Antigen discovery for vaccine development relies on identifying immunogenic proteins of *M. tuberculosis*, and many antigens have been identified using conventional and genome-wide screening approaches. While Th1 responses are generally acknowledged to be crucial for protection against *M. tuberculosis*, a TB vaccine that induced antigen-specific CD4 T-cells did not show any efficacy against *M. tuberculosis* infection in an infant efficacy trial^6^. New antigens that can activate multiple immune components, including the non-classical T-cell and antibody responses, are important for vaccine development. Recent studies have demonstrated that antibodies against different surface antigens are able to impart moderate protection against *M. tuberculosis* in preclinical animal models^7–10^. Preclinical evaluation studies in non-human primates indicate that multi-antigenic vectored vaccines, which combine latency antigens with classical early secreted antigens and resuscitation promoting factors of *M. tuberculosis*, have a strong vaccine potential. Immunization of rhesus macaques with this cytomegalovirus-based vaccine was able to impart unprecedented protection against TB disease^11^. Multi-antigenic vectored vaccination strategies have also demonstrated potential as immunotherapeutics in post-challenge mouse models^12^. The preclinical success of two distinct vaccine candidates in achieving sterilizing immunity in a proportion of animals shows that an effective TB vaccine is feasible and may elicit unique immune mechanisms of protection^11,13^. The major hindrance in developing new TB vaccines is a lack of (i) understanding of how antigens are recognized by the human immune system, (ii) identification of reliable correlates of protection, and (iii) availability of preclinical model that recapitulates human TB infection. New directions in preclinical vaccine evaluation models have the potential to complement the advancements in antigen discovery and enable the development of efficacious TB vaccines. In this review, we summarize various antigen discovery strategies for TB vaccine development. In addition, we also summarize recent developments in the in vitro and in vivo models for TB vaccine evaluation.

## Classical antigen discovery approaches and identification of culture filtrate antigens

**Fig. 1:**
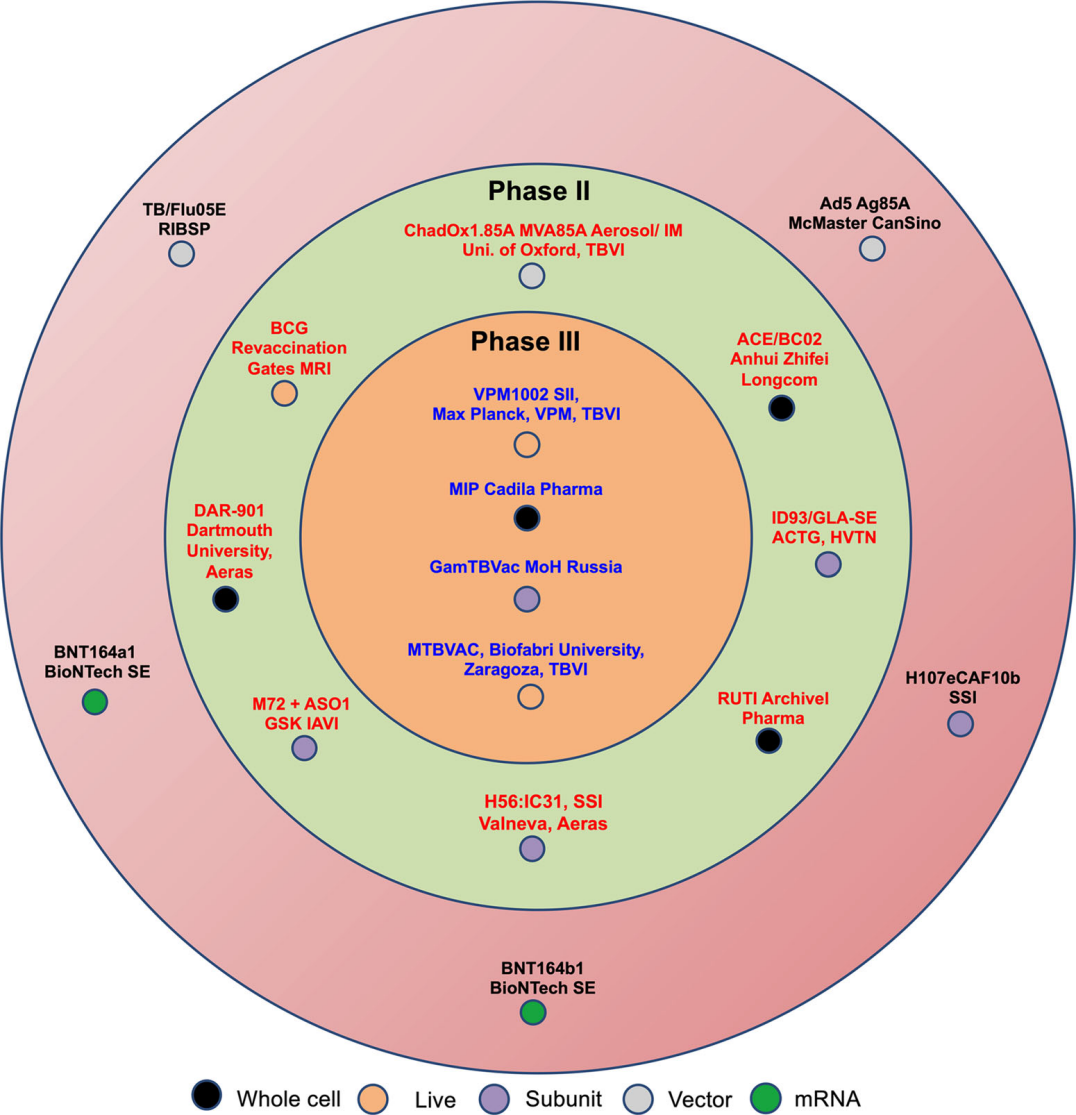
Summary of TB vaccine candidates in clinical development pipeline. Globally currently 17 vaccine candidates are being evaluated in different stages of clinical development. These candidate vaccines can be classified into five categories: viral vector based, subunit vaccines, live attenuated, mRNA or whole cell vaccines from other strains of *Mycobacterium*. These vaccines are currently being evaluated in either Phase I or II or III.

TB vaccine development has primarily focused on identifying *M. tuberculosis* secretory culture filtrate (CF) antigens. Secreted proteins are key protective antigens extensively studied as targets for T-cell induced immune responses against *M. tuberculosis*^14–16^. Studies with mice infected with virulent *M. tuberculosis* revealed the presence of specific T-cells targeting the secreted antigens of *M. tuberculosis*^15^. To identify immunogenic proteins for TB vaccine development, culture filtrate proteins (CFP) of *M. tuberculosis* were used for stimulation of peripheral blood mononuclear cells (PBMCs) from purified protein derivative (PPD)-positive human donors and T-cells derived from infected mice or guinea pigs. PBMCs and T-cells exhibited a proliferative response to *M. tuberculosis* CFP^17–20^. Immunization of mice with *M. tuberculosis* short-term culture filtrate (ST-CF), a complex mixture of secreted proteins, resulted in the generation of long-lived CD4^+^ T-cells and significant protection against *M. tuberculosis* infection (∼5.0- and ∼17.0-fold reduction in bacterial counts in lungs and spleen, respectively compared to sham immunized mice)^21^. Guinea pigs immunized with defined fractions of *M. tuberculosis* CF also showed protection against the virulent *M. tuberculosis* challenge, with reduced viable bacilli in organs and lower mortality rates compared to sham-immunized animals^22^. These studies established *M. tuberculosis* CFP as a source of immunologically important antigens that can impart protection against infection. In subsequent studies, individual immunogenic antigens of *M. tuberculosis* were identified from *M. tuberculosis* ST-CF^23,24^. Several T-cell antigens, such as antigen 85 complex proteins (Ag85A/Rv3804c, Ag85B/Rv1886c and Ag85C/Rv0129c), 6-kDa secreted antigen (ESAT-6, Rv3875), and MPT-64 (Rv1980c) were identified from *M. tuberculosis* ST-CF^19,25–35^. The components of the Ag85 complex are the most abundant secreted mycobacterial proteins which are required to synthesize mycolyl arabinogalactan (mAG) and trehalose dimycolate (TDM), important constituents of the *M. tuberculosis* cellular envelope. As a result, they are also important for disease establishment in animal models^36^. Several vaccine candidates under evaluation in clinical trials, such as H4-IC31, H56-IC31, and Crucell Ad35, include antigen 85 complex proteins as candidate antigens (Table 1, Fig. 1).

**Table 1:** Details of TB vaccine candidates in different stages of clinical trials

Vaccine/ type/ vaccination Strategy	Details	Efficacy studies in animal models (mice, guinea pigs and non-human primates)	Reference
MTBVAC/Live attenuated / Prime	A *M. tuberculosis* mutant with deletion mutations in the virulence genes *phoP* and *fadD26* encoding two major virulence factors.	**1**. Subcutaneous immunization of C57BL/6 mice with MTBVAC resulted in ～4.0-5.0-fold better protection in the lungs compared to immunization with BCG. **2**. Studies in SCID mice showed comparable safety profiles as BCG. **3**. Subcutaneous immunization of C57BL/6 and Balb/c mice with MTBVAC followed by intranasal *M. tuberculosis* infection showed similar efficacy to BCG but significantly reduced pulmonary bacterial load ( ~ 4.0-fold) in C3H mice. **4**. Revaccination of BCG-immunized guinea pigs with MTBVAC resulted in a ～150.0-fold reduction in lung bacillary loads compared to the sham-immunized group. **5**. MTBVAC vaccination in guinea pigs had ~3.0-4.0-fold reduced lung bacillary loads, compared to BCG immunized group when given 30 weeks prior to the challenge. **6**. MTBVAC vaccination was safe and showed a 3.0-fold reduction in lung bacillary loads compared to BCG-immunized newborn mice. **7**. Intradermal vaccination of adult rhesus macaques with MTBVAC resulted in significantly reduced CT scan scores compared to the BCG-vaccinated group at 12 weeks post-challenge.	Arbues et al., 2013, Aguilo et al., 2017, Clark et al., 2017, Aguilo et al., 2016, White et al., 2021.
AEC/BC02/Recombinant/ Prime-Boost	A recombinant vaccine expressing Ag85B and ESAT6-CFP10 fusion protein.	**1**. ∼200-fold reduction in bacterial burdens in lungs in latent infection model of guinea pigs compared to the saline group **2**. In a spontaneous *M. tuberculosis* relapse model, mice receiving chemotherapy and AEC/BCO2 displayed significantly lower bacterial load in the lungs and spleen (4.0-20.0-fold reduction) compared to chemotherapy-only group.	Lu et al., 2015, Guo et al., 2022, Lu et al., 2022.
Ad5-Ag85A/Viral vectored/Prime-Boost	A replication-deficient serotype 5 Adenovirus vector expressing Ag85A.	**1**. Ad5-Ag85A, when administered via the intranasal route, imparted superior protection in the lung (5.0-10.0-fold) compared to immunization of BCG in mice. **2**. Booster of BCG immunized mice with intranasal Ad5-Ag85A resulted in ∼100-fold reduction in bacterial counts post- *M. tuberculosis* challenge compared to naïve mice. **3**. 60% increased survival of BCG-immunized guinea pigs when boosted intranasally with Ad5-Ag85A. **4**. Booster of BCG-primed macaques with Ad5-Ag85A reduced lung bacterial loads by at least 100-fold compared to the non-vaccinated group. The lung bacillary loads in these animals were reduced by 5.0- and 100.0-fold in the right and left lung, respectively, compared to BCG-immunized animals, depending on the route of vaccination.	Wang et al., 2004, Santosuosso et al. 2006, Xing et al., 2009, Jeyanathan et al., 2015.
ChAdOx185A-MVA85A/Viral Vectored/Prime Boost	A simian adenovirus expressing Ag85A.	**1**. Booster of BCG-immunized mice with ChAdOx1.85A followed by MVA85A significantly reduced bacterial loads by ~5.0-fold at 4 weeks post *M. tuberculosis* challenge.	Stylianou et al., 2015.
RUTI®/Fragmented *M. tuberculosis*/Immunotherapy	Detoxified liposomal fragments of *M. tuberculosis*.	**1**. Administration of RUTI after a short period of chemotherapy in latent tuberculosis infection models in mice and guinea pigs showed reduced lung bacillary loads compared to chemotherapy alone. **2**. SCID mice receiving serum from mice inoculated with RUTI were protected against *M. tuberculosis* reaction after chemotherapy. **3**. RUTI administration enhanced the efficacy of standard anti-TB chemotherapy as compared to chemotherapy alone in mice. **4**. Short-term prophylactic effect of RUTI in mice model was less effective than BCG. In the long-term prophylactic model, RUTI and BCG administration imparted comparable protection in mice lung tissues. **5**. The survival of guinea pigs receiving prophylactic vaccination with RUTI was similar to BCG-vaccinated animals.	Cardona et al., 2005 and 2006, Guirado et al., 2008, Guirado et al., 2006, Soldevilla et al., 2023, Vilaplana et al., 2011.
H56:IC31/adjuvanted subunit/Prime-Boost	A fusion protein of *M. tuberculosis* antigens (Ag85B, ESAT-6 and Rv2660c) formulated in the adjuvant IC31.	**1**. H56-vaccination resulted in ∼5.0-fold better protection in lungs compared to BCG immunized mice at 12- and 24-week post-challenge. **2**. Booster of BCG immunized primates with H56 reduced clinical symptoms and prevented disease reactivation. **3**. BCG/H56 immunization imparted better protection than BCG alone in the non-human primate model.	Aagaard et al.,^76^, Lin et al.,^77^.
ID93 + GLA-SE/Adjuvanted subunit/Prime-Boost	A recombinant fusion-protein of *M. tuberculosis* antigens (virulence-associated Rv2608, Rv3619, Rv3620, and latency-associated Rv1813).	**1**. Immunization with ID93/GLA-SE resulted in ∼3.0-fold reduced bacterial numbers in mice infected with drug-susceptible (lungs and spleens) and multi-drug-resistant strain (lungs) in comparison to saline-immunized mice. **2**. Boosting of BCG with ID93 + GLA-SE significantly increased the survival of *M. tuberculosis*-infected guinea pigs in comparison to BCG-immunized animals. **3**. Immunization of non-human primates with ID93 post-INH/RIF treatment significantly reduced bacterial numbers in comparison to the drug-treatment group only.	Bertholet et al., 2010, Coler et al., 2013.
TB/FLU-05E/Viral vectored/Prime-Boost	A recombinant attenuated influenza vector (Flu/THSP) co-expressing truncated NS1 protein NS1(1–124) and a full-length TB10.4 and HspX proteins of *M. tuberculosis*.	**1**. Mucosal immunization with TB/FLU-05E provided **≥** 3.0-fold protection in the lungs and spleen of mice, which was comparable to BCG. **2**. TB/FLU-05E is safe and immunogenic in mice and guinea pigs.. **3**. Boosting BCG-immunized guinea pigs with TB/FLU-05E significantly enhanced BCG’s protective efficacy in lungs and spleens. **4**. Boosting BCG with intranasal immunization of TB/FLU-05E provided greater protection than BCG in *M. tuberculosis*-infected mice.	Sergeeva et al., 2021, Vesilyev et al., 2021.
DAR-901 booster/Whole cell/Prime-Boost	Heat inactivated whole cell *Mycobacterium obuense*.	**1**. BCG primed mice with DAR-901 boosters provided comparable protection to BCG immunized animals in lungs and spleens.	Lahey et al., 2016.
H107e/CAF10b/ Subunit vaccine	TB subunit vaccine incorporating *M. tuberculosis* antigens (PPE68, ESAT-6, EspI, EspC, and EspA, MPT64, MPT70, and MPT83) in a novel liposomal Th1/Th17 inducing adjuvant.	**1**. BCG and H107 co-administration in mice induced superior short and long-term protection compared to BCG alone against *M. tuberculosis* challenge.	Woodworth et al., 2021.
M72/AS01E/Adjuvanted subunit/Prime-Boost	An immunogenic fusion protein (M72) derived from two *M. tuberculosis* antigens (Mtb32A and Mtb39A) and the adjuvant AS01E.	**1**. The levels of protection in mice vaccinated with BCG and boosted with polyprotein Mtb72F-AS02A was lower but not significantly different to BCG immunized mice. **2**. Co-administration of BCG with Mtb72F-AS02A significantly improved the survival of *M. tuberculosis* infected guinea pigs compared to BCG immunized animals. **3**. In cynomolgus macaques boosting BCG with Mtb72F-AS02A resulted in superior protection than BCG immunized non-human primates, as measured by survival.	Brandt et al., 2004. Reed et al., 2009,
VPM1002/Recombinant/ Prime	A recombinant BCG mutant harboring deletion in urease C and expressing listeriolysin O	**1**. Immunization of mice with VPM1002 resulted in ~10.0-fold better protection against *M. tuberculosis* infection compared to BCG immunized group. **2**. In a post-exposure mouse model, immunization of mice with VPM1002 reduced lung bacterial loads by 4.0-fold compared to BCG immunized mice. **3**. Safety of VPM1002 has been established in immunodeficient mice models, guinea pigs, rabbits, and non-human primates.	Grode et al., 2005, Kaufmann et al., 2014, Gengenbacher et al., 2016.
BCG revaccination/Live attenuated/Prime-Boost	BCG revaccination.	In C57BL/6 neonates, multiple doses of BCG reduced the bacterial burden at early time points post *M. tuberculosis* challenge compared to single BCG immunization. However, some preclinical studies suggest that revaccination with BCG does not enhance protection.	Li et al., 2012, Buddle et al., 2003.
*Mycobacterium Indicus Pranii* (MIP)/Immuvac/Whole cell/Immunotherapy	Heat-inactivated whole cell vaccine.	**1**. Immunotherapy of guinea pigs with MIP as an adjunct reduced lung and splenic bacterial load (by ~ 5.0-10.0 folds) and organ pathology as compared to chemotherapy alone. **2**. Adoptive transfer of airway-resident T cell populations from mice intranasally immunized with MIP confers protection in naïve mice against *M. tuberculosis* challenge (by ~ 5.0-fold compared to sham immunized controls). **3**. Prophylactic immunization of guinea pigs with MIP resulted in 10.0-fold reduced bacterial loads compared to BCG immunized animals. **4**. MIP given as a prophylactic booster to BCG significantly reduced the lungs and splenic bacterial burdens post *M. tuberculosis* challenge in guinea pigs and mice compared to BCG immunized group.	Gupta et al., 2012, Gupta et al., 2019, Gupta et al., 2012, Saqib et al., 2016.
GamTBvac/ Subunit vaccine/Prime-Boost	A recombinant vaccine containing *M. tuberculosis* antigens: Ag85A and ESAT6-CFP10 fusion protein, fused with a dextran-binding domain (DBD) from *Leuconostoc mesenteroides*. These fusions are formulated with the adjuvant containing Dextran 500 kDa and DEAE-Dextran 500 kDa covered with CpG oligonucleotides.	**1**. Boosting of BCG with GamTBvac resulted in comparable protection to BCG alone in mice and guinea pigs infected with *M. tuberculosis* via intravenous or aerosol route**. 2**. Prime-boost immunization significantly enhanced the survival of guinea pigs infected with *M. tuberculosis* than BCG alone.	Tkachuk et al., 2017.

To identify T-cell antigens present in the ST-CF of *M. tuberculosis*, electroeluted protein fractions were used to stimulate T-cells derived from human donors. T-cells obtained from donors with active TB showed robust IFN-γ responses against the low-molecular-mass (<10-kDa) protein fraction^18^. In C57BL/6 mice, ESAT-6 found in the ST-CF, along with Ag85B, were primary antigenic targets for memory effector cells involved in the recall of memory immune recall response^37^. ESAT-6, a pore-forming toxin necessary for *M. tuberculosis* pathogenicity, has shown great promise as a T-cell antigen for TB vaccine development^37^. ESAT-6 is a component of several TB vaccine candidates currently evaluated in clinical trials, such as H1-IC31, H56-IC31, and TB/FLU-04L (Table 1, Fig. 1). Another promising low molecular mass antigen found in the *M. tuberculosis* ST-CF is CFP-10 (Rv3874) which co-transcribes with ESAT-6^38^. ESAT-6 and CFP-10 are encoded by the region of difference 1 (RD1) and secreted by the ESAT-6 system 1 (ESX-1) secretion system^39^. Regions of differences (RDs) are genomic segments identified by comparative genomic analysis that are present in the *M. tuberculosis* complex but absent from most BCG strains and several non-tuberculosis mycobacteria (NTM)^40^. CFP-10 is being assessed as a component of the AEC-BCO2 vaccine currently in clinical trial (Table 1, Fig. 1). Several antigens encoded by RD1 region, such as CFP-10 and ESAT-6 have also been translated into diagnostic tests for detecting *M. tuberculosis* infection. ESX-1 dependent ESAT-6 secretion in recombinant BCG (rBCG:ESX-1) is associated with enhanced immunogenicity and protection against *M. tuberculosis* in animal models. However, the major disadvantages associated with rBCG:ESX-1 strain is its increased virulence and prolonged persistence in immunocompromised mice^41,42^. Uncoupling of ESAT-6 from detrimental effects of ESX-1 dependent secretion was attained by fusing ESAT-6 with secretion signal for the mycobacterial type VII secretion pathway protein PE25, resulting in a new strain, rBCG::ESAT-6-PE25SS. This recombinant BCG strain secretes full-length ESAT-6 via the ESX-5 secretion system^43^. The authors showed that immunization of C57BL/6 mice with rBCG::ESAT-6-PE25SS induces cytosolic contact, generates ESAT-6-specific T-cells and enhances the protective efficacy of BCG (as observed by a reduction in lung pathology and bacterial burden) following *M. tuberculosis* infection^43^. Further, in comparison to rBCG:ESX-1, intratracheal immunization and intravenous injection with rBCG::ESAT-6-PE25SS were associated with reduced virulence and persistence in immunocompetent and immunocompromised mice, respectively^43^. In addition to Ag85 complex proteins, ESAT-6, and CFP-10, several other immunodominant antigens, such as 38 kDa antigen (Rv0934) and MPT51 (Rv3803c), were identified in the pre-genomic era^44,45^. Coler et al. separated *M. tuberculosis* CFP into 16 fractions and evaluated immunogenicity using PBMCs from PPD-positive healthy volunteers^46^. This study identified MTB8.4 (Rv1174c), which stimulated PBMCs from PPD-positive healthy donors but not PPD-negative donors and elicited Th1 response in the murine immunogenicity studies^46^. When administered as an adjuvanted recombinant protein or as a DNA vaccine, MTB8.4 induced robust CD4^+^ helper T-cell and cytotoxic T-cell responses, and the level of protection was comparable to BCG-immunized mice (~10.0-fold reduction in both lungs and spleens)^47^. In a large-scale proteomics approach, CFP and cytosolic proteins of *M. tuberculosis* were fractionated into 335 and 299 protein fractions, respectively, and used to identify antigens that induce high IFN-γ production from splenocytes of *M. tuberculosis* infected mice^48^. A total of 30 proteins were identified as immunodominant T-cell antigens, and more than half of these proteins were unidentified T-cell antigens. These observations demonstrated the application of large-scale proteomics in the identification of immunodominant T-cell antigens^48^.

## Discovery of dormancy antigens and resuscitation promoting factors as promising vaccine target antigens

*M. tuberculosis* considerably alters its antigenic repertoire during different stages of infection^49^. During in vivo infection, *M. tuberculosis* multiplication is typically controlled by the host immune system and enters dormancy with reduced or no metabolic activity^50^. In vitro dormancy models capture different aspects of the hypoxic environment encountered by the dormant bacilli in vivo^51,52^. Microarray studies have identified >100 *M. tuberculosis* genes that are differentially expressed in response to hypoxic conditions^53^. The 16-kDa α-crystallin-like small heat shock protein (HspX/Acr, Rv2031c) is one of the most prominent latency antigens, with its expression markedly increased under oxygen-limiting conditions and in stationary phase cultures of *M. tuberculosis*^51,54,55^. HspX is required for the intracellular growth of *M. tuberculosis* in macrophages and is involved in cell wall thickening under hypoxic environments^56^. HspX has demonstrated robust immunogenicity in animal models and humans^57–61^. Based on these observations, HspX has been used as a candidate vaccine antigen in multiple TB vaccine constructs^62–72^. The analysis of the *M. tuberculosis* transcriptome and proteome in an in vitro nutrient starvation model resulted in the identification of proteins necessary for adaptation to nutrient starvation^73^. Among these starvation-induced proteins, Rv2660, (a conserved hypothetical protein) was the most upregulated protein^73–75^. Rv2660c is currently being evaluated in the clinical trial as a component of the multistage TB vaccine candidate H56 (Table 1, Fig. 1)^76^. The H56 vaccine is a protein vaccine that combines the latency-associated antigen Rv2660c, with early antigens Ag85B and ESAT-6 formulated in a cationic adjuvant (CAF01)^76^. In a pre-exposure mouse model, boosting BCG with H56 resulted in the induction of vaccine-specific polyfunctional CD4^+^ T-cell response and a significant reduction (~7.0-fold) in lung bacterial load compared to immunization with BCG alone^76^. Vaccination with H56 post-*M. tuberculosis* exposure protected against latent TB reactivation, and boosting BCG with H56 prevented latent reactivation in cynomolgus macaques^76,77^. To identify human T-cell antigens suitable for subunit TB vaccine development, Bertholet et al. screened 94 *M. tuberculosis* proteins belonging to either PE/PPE or ESX family or proteins involved in growth in macrophages, hypoxia, secretion, or membrane association for IFN-γ recall responses using PBMCs from healthy subjects exposed to *M. tuberculosis*^78^. Based on these screening results, 48 antigens were further tested as adjuvanted subunit vaccines in the murine *M. tuberculosis* aerosol infection model. Three secreted proteins, two PE/PPE proteins, and one hypoxia-associated protein showed >0.3 log_10_ reduction in lung CFU compared to sham-immunized mice. Rv1813c (hypoxia-associated), Rv3620 (ESX family), and Rv2608 (PE/PPE family) were combined in a multi-antigen subunit vaccine formulation with CpG adjuvant. Immunization of mice with this multi-antigen subunit vaccine reduced lung bacterial loads compared to adjuvant only control by ∼5.0-fold, and the observed protection was comparable to BCG-vaccinated mice^78^. The same three antigens, Rv1813c, Rv2608, and Rv3620, together with Rv3619, are included as a fusion protein in the subunit vaccine construct ID93 combined with adjuvant GLA-SE and are currently in the TB vaccine trial pipeline^79^ (Table 1, Fig. 1).

*M. tuberculosis* induces the expression of an array of latency-associated antigens belonging to the 48-gene-DosR regulon as an adaptive response to dormancy^75,80^. *M. tuberculosis* expresses the DosR regulon under low oxygen and nitric oxide conditions^80^. Several studies have demonstrated the immunogenicity and vaccine potential of the dormancy regulon-associated antigens^65,81–86^. In a study evaluating human T-cell responses to 25 strongly expressed proteins of the DosR regulon, the majority of the antigens generated strong IFN-γ response among tuberculin skin test (TST) positive individuals with latent TB, in comparison to active TB patients, indicating their potential protective role in infected individuals^86^. Notably, Rv1733c, Rv2029c, Rv2627c, and Rv2628, were predominantly recognized by TST-positive individuals and have emerged as promising antigenic targets^86^. In concordance, long-term latently infected individuals showed the presence of mono- and polyfunctional cytokine-producing CD4^+^ and CD8^+^ T-cells specific for DosR encoded antigens Rv1733c, Rv2029c and Rv2031c^87^. DosR-regulon-encoded antigen Rv2628 was strongly recognized by individuals with a history of TB infection (≥ three years before study enrolment)^88^. Immunization of mice with DNA vaccines encoding Rv2031c (HspX) and Rv2626c induced robust Th1 response, and several T-cell epitopes were mapped on these antigens^65^. Rv1733c encodes a membrane protein and is the most frequently recognized DosR regulon encoded protein in *M. tuberculosis* exposed household contacts from three high TB burden populations in Africa^82^. Immunization of mice with a synthetic long peptide (SLP) derived from Rv1733c induced a potent T-cell response and improved the efficacy of BCG, resulting in a ~8.0-fold reduction in lung bacillary load compared to BCG-immunized mice (~5.0-fold reduction)^83^. Mice immunized with recombinant BCG expressing Rv1733c along with Rv2659c (latency antigen), Rv3407 (reactivation antigen), and membrane-perforating listeriolysin (*hly*) significantly induced IFN-γ response in splenocytes and imparted 10.0-fold better protection (reduced bacterial burdens in lungs) compared to BCG-immunized mice against *M. tuberculosis* Beijing/W challenge^84^. The immunomodulatory properties of dormancy antigens as Toll-like receptor (TLR) agonists have also attracted attention as adjuvants for developing subunit vaccines. The use of HspX as a TLR agonist in adjuvant-based tumor immunotherapy has been reported^89^. Recent studies showed that dormancy-associated *M. tuberculosis* antigens such as Rv2627c, Rv2628, and Rv2659c induce innate and adaptive immune responses through TLR activation^90,91^. The adjuvant-like properties of these antigens could be harnessed for the rational design of multi-epitope subunit vaccines.

Resuscitation-promoting factors are growth-promoting proteins that can reactivate the growth of dormant bacteria and are immunogenic^92–95^. The genome of *M. tuberculosis* encodes for five resuscitation-promoting factors *(rpf)*, namely Rv0867c *(rpfA)*, Rv1009 *(rpfB)*, Rv1884c *(rpfC)*, Rv2389c *(rpfD)* and Rv2450c *(rpfE)*. Among these, RpfB has emerged as a promising vaccine candidate antigen^96,97^. Over the past decade, a significant number of multi-antigenic and multi-phasic TB vaccine candidates have been developed, aiming to combine early antigens with Rpfs to prevent early infection and TB reactivation^11,12,98–100^. MVATG18598, an MVA-based multi-antigenic vaccine, combines ten immunogenic antigens from various stages of infection, including early antigens ESAT-6, CFP-10, Rv0287, TB10.4 (Rv0288), and Ag85B, late antigens Rv2626c, Rv1813c, and Rv3407 and resuscitation phase antigens RpfB and RpfD^12^. In a post-exposure mouse model of *M. tuberculosis* infection, MVATG18598 vaccination and standard antibiotic regimen were associated with long-lasting cellular and humoral response and greater reduction in the bacterial burden compared to chemotherapy alone^12^. Five antigens used in MVATG18598 were also used in a rhesus cytomegalovirus-based TB vaccine candidate (RhCMV/TB). Immunization with RhCMV/TB-9 expressing Rv3407 along with ESAT-6, Ag85A, Ag85B, Rv1733, Rv2626c, RpfA, RpfC, and RpfD showed exceptional long-term efficacy against infection with *M. tuberculosis* Erdman strain and 41% of vaccinated rhesus macaques showed no TB disease compared to unvaccinated macaques^11^. In the same study, RhCMV/TB vaccine construct expressing *M. tuberculosis* polyprotein of six antigens (ESAT-6, Ag85A, Rv3407, Rv2626c, RpfA, and RpfD) could recapitulate the sterilizing vaccine immunity of RhCMV/TB-9 antigen vector combination^11^. These observations challenge the role of some immunodominant antigens in protective immunity against TB and also highlight the potential benefit of combining antigens in designing an effective TB vaccine^11^.

Heparin‐binding haemagglutinin (HBHA), an extrapulmonary dissemination factor, induces strong cellular immunity in individuals with latent TB infection (LTBI) but not in those with active TB^101,102^. HBHA, a cell surface protein, is involved in the binding of mycobacteria to epithelial cells facilitating the dissemination of infection to extrapulmonary sites and contributing to TB pathogenesis^103,104^. Studies have investigated HBHA as an antigen of diagnostic and protective value against TB^105–108^. When administered with adjuvant, immunization with purified HBHA protein resulted in an approximately 5.0-fold reduction in lung bacterial burden compared to the adjuvant control mice 28 days post-challenge^105^. Subcutaneous administration of rHBHA improved the effectiveness of BCG in the spleens and lungs of mice challenged with *M. tuberculosis* via the intranasal route and only the spleens of aerosol-infected mice^109^.

## *M. tuberculosis* genomic expression library

Serological screening of *M. tuberculosis* genome expression libraries has been used to identify several promising T-cell antigens. Young et al. constructed a genomic expression library representing the complete *M. tuberculosis* genome in the λgt-11 expression vector^110^. Several studies have used this λgt-11 *M. tuberculosis* genomic expression library to screen TB patient sera, rabbit polyclonal sera, and murine monoclonal antibodies to identify antigenic proteins or epitopes^44,110–119^. *M. tuberculosis* genomic expression library screening has been used to identify immunogenic components of ST-CF, such as the 96 amino acid antigen TB10.4^120^. The simultaneous expression of antigenic proteins TB10.4, Ag85A and Ag85B in a recombinant BCG-based vaccine expressing perfringolysin O (AFRO-1) yielded an enhanced immune response in infected mice and guinea pigs compared to perfringolysin O expressing BCG strain (AERAS-401)^121^. The expression of perfringolysin O in BCG leads to the lysis of the endosomal membrane and increased antigen presentation^122^. Mice immunized with AFRO-1 and infected with the highly pathogenic *M. tuberculosis* HN878 survived longer than parental BCG_1331_ strain^121^. TB10.4 is a component of the vaccine constructs H4 + IC31, H1 + IC31, and Crucell Ad35 currently in the TB vaccine clinical trial pipeline (Table 1, Fig. 1). However, recent studies have proposed that *M. tuberculosis* uses TB10.4 as a “decoy” protein by inducing immunodominant antigen-specific CD8 T cells that poorly recognize *M. tuberculosis-* infected macrophages, and therefore, cannot mediate optimal protection^123,124^. Such findings highlight the critical role of choice of antigens in developing new TB vaccine candidates.

Screening of the H37Ra genomic expression library with rabbit antiserum raised against the CFP of *M. tuberculosis* Erdman identified two secreted serine proteases MTB32A (Rv0125) and MTB32B (Rv0983) as immunogenic antigens^125^. These antigens have been evaluated in a clinical trial with the adjuvant AS01E as a subunit vaccine M72/AS01E (Table 1, Fig. 1). Phase IIb trial studies of M72/AS01E have shown promising results. Immunization with M72/AS01E imparted ~50% protection (95% CI, 1.8 to 74.1) against active pulmonary TB disease in human immunodeficiency virus (HIV)–negative adults with latent *M. tuberculosis* infection^126^. Dillon et al. used pooled sera from TB patients for serological screening of the *M. tuberculosis* genomic expression library and identified MTB39A (Rv1196) as an immunogenic antigen. The authors showed that MTB39A elicited T-cell proliferation and IFN-γ production in PBMCs of PPD-positive healthy individuals^127^. MTB39A belongs to the PE/PPE family and is present in *M. tuberculosis* lysate but not in CFP, providing evidence that potent T-cell antigens are not exclusively present in CFP. Immunization of C57BL/6 mice with DNA vaccine encoding MTB39A reduced the lung bacillary load by 5.0-fold compared to sham-immunized mice. However, protection was lower compared to BCG-vaccinated mice (10.0-fold reduction in *M. tuberculosis* burden compared to sham-immunized animals)^127^.

Serological screening of *M. tuberculosis* expression libraries has limited potential since immunogenic T-cell antigens may not necessarily produce robust antibody responses and, thus, may not be captured by this approach. In contrast, T-cell expression cloning is a technique used for the direct identification of mycobacterial antigens involved in disease control^127–129^. Alderson et al. developed an expression cloning approach using *M. tuberculosis*-specific protective CD4^+^ T-cell line generated from healthy PPD-positive volunteers (non-BCG-vaccinated)^129^. This study identified MTB9.9A (Rv1793), which was recognized by T-cells from healthy PPD-positive individuals but not PPD-negative volunteers^129^. Skeiky et al. generated anti-*M. tuberculosis*-CFP-specific CD4^+^ T-cell line from infected C57BL/6 mice to identify antigens linked with the early control of *M. tuberculosis* infection^128^. This study identified MTB41 (Rv0915c) belonging to the PE/PPE family of proteins as a candidate antigen for TB vaccine development. Mice immunized with a DNA vaccine expressing MTB41-induced antigen-specific CD4^+^ and CD8^+^ responses, and this vaccination imparted protection comparable to BCG (~8.0–10.0-fold reduction in lung bacillary load of DNA vaccine immunized and BCG-immunized C57BL/6 mice compared to vector immunized mice)^128^. However, although expression cloning is a promising method, its application is limited to the identification of protein antigens. Additionally, the influence of post-translational modifications arising from heterologous expression on the immunogenicity of antigens remains a limitation^130^.

## In silico approaches

Computational vaccinology has accelerated vaccine design by enabling epitope prediction and selection of immunogenic antigens at a low cost^131^. Numerous studies have employed genome-wide screening and reverse vaccinology to identify uncharacterized immunogenic epitopes and proteins without any antigen bias. The availability of *M. tuberculosis* genome sequence has facilitated the identification of potential candidate vaccine antigens using comprehensive data mining and in silico tools^131,132^. Zvi et al. constructed a bioinformatics genome analysis pipeline and identified 45 top-ranking candidate antigens covering various stages of TB disease^133^. Among the antigens discovered by Zvi et al., at least 17 candidate antigens have been evaluated in clinical trials and preclinical models. The majority of the 45 top-ranked antigens included DosR-regulated proteins, reactivation/resuscitation proteins, cell wall proteins, classical vaccine candidates, and conserved hypothetical proteins. Based on the list of 45 top-ranked antigens, rBCG AERAS-407 was designed to overexpress (i) classical antigens such as Ag85A and Ag85B, (ii) dormancy or resuscitation antigens including RpfA, RpfC, and RpfD, and (iii) *M. tuberculosis* specific antigen Rv3407. Compared to BCG-immunized mice, immunization with rBCG AERAS-407 resulted in a more pronounced antibody and cellular immune response. rBCG AERAS-407 immunized mice showed a greater reduction in viable bacilli count in lungs and spleens compared to BCG vaccinated mice against *M. tuberculosis* Erdman at ten weeks post-challenge^134^.

In a genome-wide approach, five complete *M. tuberculosis* genomes and 16 draft assemblies were screened in silico for 15-mer peptides predicted to bind to the most frequently expressed human leukocyte antigen (HLA) class II alleles^135^. The peptides were subsequently analyzed for ex vivo IFN-γ production by PBMCs from LTBI donors. A total of 82 antigens were identified that are frequently recognized by LTBI donors. Among these, 34 antigens have not been previously classified as T-cell antigens^135^. Furthermore, several of these antigens were uniformly recognized by LTBI donors from different geographical locations, indicating their potential use in developing vaccines targeting diverse populations^136^. Using in silico epitope prediction tools, De Groot et al. identified class II MHC binding epitopes from known secreted antigens such as MPT64, Ag85B, MPT70, MPT63, 38 kDa protein, 14 kDa, 16 kDa, 19 kDa, and 32 kDa protein as well as from ORFs containing signal sequences^137^. TB 001, a DNA vaccine construct overexpressing 24 selected epitopes, stimulated epitope-specific T-cell responses in HLA DRB1*0101 transgenic mice expressing the human MHC haplotype of class II antigens^137^. IKEPLUS is a genetically modified *M. smegmatis* strain expressing ESX-3 type VII secretion system of *M. tuberculosis*, and immunization of mice with IKEPLUS resulted in strong bactericidal CD4^+^ T-cell responses and prolonged survival of C57BL/6 mice after *M. tuberculosis* challenge^138^. A synthetic peptide library of 880 nonoverlapping peptides was constructed by in silico prediction of possible 15-mer peptides from the genome sequences of *M. smegmatis* and *M. tuberculosis* to examine the range of CD4^+^ T-cell epitopes in IKEPLUS-immunized mice. Screening of the peptide library by an IFN-γ ELISPOT assay using CD4^+^ T-cell isolated at two weeks post-immunization from spleens of IKEPLUS immunized C57BL/6 mice showed that mycobacterial ribosome was a major target of CD4^+^ T-cell^139^. The study identified a specific immunogenic peptide within the mycobacterial ribosomal large subunit protein RplJ (Rv0651). Compared to BCG-immunized mice, CD4^+^ T-cells from IKEPLUS-immunized mice responded significantly to the 15-mer RplJTB_146 –160_ epitope. In another study, boosting of BCG primed immunity with the *M. tuberculosis* RplJ protein resulted in significantly reduced lung pathology and bacterial burden in mediastinal lymph nodes compared to naïve or BCG-immunized mice^140^. These findings suggest that ribosomal proteins are potential sources of antigenic targets that are recognized by protective CD4^+^ T-cells.

In addition to CD4^+^ T-cells, HLA class I-restricted CD8^+^ T-cells play an essential role in controlling *M*. *tuberculosis* infection and mediating optimal host response in small animal models, non-human primates, and humans^141^. However, conventional antigen discovery approaches have only identified a few CD8^+^ T-cell epitopes. The repertoire of CD8^+^ T-cell epitopes identified in *M. tuberculosis* has increased as a result of in silico epitope prediction approaches. In one of the first genome-wide screens to identify CD8^+^ T-cell epitopes, all 3924 *M. tuberculosis* ORFs were searched for HLA-B*35 restricted CD8^+^ T-cell epitopes, common HLA-B type in West Africa^142^. Among the 479 predicted CD8^+^ T-cell epitopes, 13 most promising epitopes belonged to uncharacterized mycobacterial antigens and elicited a CD8^+^ T-cell response in healthy BCG-vaccinated donors naturally exposed to mycobacteria^142^. Using genome-wide in silico epitope prediction tools and immunological screening, Tang et al. identified CD8^+^ T-cell epitopes presented by HLA class I supertypes that account for 80% of the human population^143^. The antigens that were either secretory or being evaluated in various TB vaccine candidates had the highest frequency of predicted CD8^+^ T-cell epitopes. Following immunological screening, a number of new *M. tuberculosis* peptides likely to be presented by CD8^+^ MHC class-I were identified in healthy PPD-positive donors. In individuals with cured TB disease, a set of epitopes was shown to trigger mono-, dual-, or triple-cytokine–producing CD8^+^ T-cell responses, indicating the polyfunctional nature of these *M. tuberculosis* peptide-specific CD8^+^ T-cells^143^. Similar epitope prediction in silico tools have been used to identify promiscuous epitopes in dormancy antigens^144^. Promiscuous epitopes are expected to have a broader population coverage since they are predicted to bind ten or more HLA alleles. Several of the promiscuous epitopes are conserved among different *M. tuberculosis* strains and have a larger population coverage than ESAT-6 and CFP-10^144^. In a T-cell-focused genome-wide search of immunodominant CD8^+^ T-cell epitopes, Lewinsohn et al. identified a set of 389 unique *M. tuberculosis* proteins likely to possess CD8^+^ T-cell epitopes^145^. Further, a 15-mer synthetic peptide library was synthesized, and this peptide library (representing 10% of the *M. tuberculosis* proteome) was screened with CD8^+^ T-cells isolated from LTBI or active TB donors. This study identified three CD8^+^ T-cell antigens, EsxJ (Rv1038c), PE9 (Rv1088), and PE_PGRS42 (Rv2487c) as promising vaccine antigens^145^.

Mycobacterial antigens are presented by classical as well as non-classical HLA molecules. Unconventional HLA molecules such as HLA-class 1b exhibit limited polymorphism. HLA class 1b molecules such as HLA-E present *M. tuberculosis* epitopes triggering CD8^+^ effector T-cell activation. HLA-E expression is not affected by HIV co-infection, which is highly prevalent in TB-endemic countries^146,147^. In the first genome-wide effort to identify CD8^+^ T-cell peptide epitopes presented by human HLA-E, in silico tools were used to scan the *M. tuberculosis* genome for putative HLA-E binding peptide epitopes^148^. This study identified 69 potential HLA-E epitopes and most of the peptides displayed CD8^+^ T-cell proliferation in PBMCs derived from PPD-positive donors and BCG immunized infants^148^. These CD8^+^ T-cell recognizing peptides presented by HLA-E represented a new population that exerts cytolytic functions, activates B-cells, and produces Th2 cytokines instead of Th1 cytokines^149,150^.

## Other genome-wide approaches

Unbiased antigen discovery approaches such as genome-wide gene expression studies have identified genes that are differentially expressed during in vivo infection with *M. tuberculosis*^151^. A set of in vivo-expressed *M. tuberculosis* genes (IVE-TB) genes was selected based on their real-time in vivo expression in the lungs of genetically related mice strains representing a spectrum of susceptibility to *M. tuberculosis* infection^151^. Several IVE-TB genes, such as Rv1363c, Rv1956, Rv2034, Rv2324, Rv2380c, Rv3353c, and Rv3420c, were identified to be highly immunogenic as measured by antigen-specific IFN-γ production by mice splenocytes. These IVE-TB antigens were also recognized by immune cells from TST-positive individuals but not recognized by cells from healthy controls^151^. IVE-TB genes also induced strong multifunctional T-cell responses in PBMCs isolated from LTBI donors^151^. Rv2034, implicated in stress response and regulation of lipid metabolism, has been evaluated as a vaccine candidate in mice and guinea pigs^152^. Rv2034 protein was highly immunogenic in HLA-DR transgenic mice and elicited cellular and humoral immune responses. Rv2034 when administered as a fusion protein along with Ag85B and ESAT-6 (H1-Rv2034/CAF09) showed a significant reduction of bacillary load in the lungs and spleen of *M. tuberculosis* infected guinea pigs by 13.0- and 436.0-fold, respectively, relative to the unvaccinated group. The levels of protection were comparable to those observed in BCG-vaccinated guinea pigs^152^. A subsequent study analyzed the expression of 2068 *M. tuberculosis* genes to identify highly upregulated IVE-TB genes during the early and late stages of infection^153^. In this study, gene expression data from the lungs of resistant and susceptible mice infected with *M. tuberculosis* identified 194 genes with altered expression. The most promising genes were further shortlisted using the following criteria: (i) top 15% genes expressed during the last six weeks of *M. tuberculosis* infection, (ii) hyper conservation and the presence of HLA class Ia and II binding epitopes, and (iii) the presence of homologs in *M. leprae*^153^. A total of 48 out of these 194 differentially expressed IVE-TB genes were recombinantly expressed and evaluated for immunogenicity using PBMCs from double responders (ESAT-6/CFP-10 positive and PPD-positive), single responders (ESAT-6/CFP-10 positive or PPD-positive) and non-responders (ESAT-6/CFP-10 negative and PPD-negative) to *M. tuberculosis*. Among these, 29 IVE-TB proteins triggered a cytokine response from PBMCs from *M. tuberculosis* exposed individuals, and nine of these antigens (Rv3615, Rv2029, Rv3353, Rv1733, Rv0826, Rv2215, Rv1791, Rv2873, and Rv2626c) triggered the secretion of cytokine response (IP-10, TNF-α, and IL-17) without induction of IFN-γ response^153^. Another study was performed in three different mice strains, including susceptible (C3HeB/FeJ) and resistant (C57BL/6J and Balb/c) murine strains, to identify IVE-TB-antigens that are recognized by T-cells following *M. tuberculosis* infection or BCG immunization^154^. This study identified 11 IVE-TB-antigens that were recognized in both TB-resistant and susceptible mice strains. These antigens induced IFN-γ, TNF-α, and IL-17 across different organs in resistant and susceptible mice. In addition, three IVE-TB antigens (Rv0470, Rv1733, and Rv3616) were strongly recognized by *M. tuberculosis* infected as well as BCG-immunized C3HeB/FeJ mice and induced strong IFN-γ response, suggesting their potential for use in prime-boost approach^154^.

Genome-wide antigen discovery efforts have also increased the repertoire of CD8 immunodominant antigens. IFN-γ ELISPOT assay-based screening of synthetic peptide library against human CD8^+^ T cells identified several immunodominant CD8 T-cell antigens. Among these, PPE15 (Rv1039c), PPE51 (Rv3136), PE12 (Rv1172c), and PE3 (Rv0159c) have been evaluated as potential candidate antigens for developing TB subunit vaccines^155–157^. In addition to being immunodominant CD8 T-cell antigens, these antigens also induced strong CD4^+^ T-cell responses in TB patients and individuals with LTBI. In subsequent studies, these antigens have been incorporated in a replication-deficient chimpanzee adenovirus (ChAdOx1), and protective efficacy was analyzed following intranasal vaccination in murine *M. tuberculosis* challenge experiments^157^. ChAdOx1.PPE15 and ChAdOx1.PPE51 reduced the lung bacillary load compared to the naïve control group (by approximately 10.0-fold), but the protection afforded was less compared to the BCG-immunized group. However, the lungs and splenic bacillary load in the BCG-immunized group was further decreased by 3.0-fold and 5.0-fold, respectively, upon booster with ChAdOx1.PPE15.

## Recent developments in antigen discovery

Recent advancements in single cell T-cell receptor sequencing technologies (scTCR-seq) and improved in silico analytical tools such as GLIPH2 (algorithm to cluster TCRs that recognize the same epitope) have allowed unprecedented throughput and efficiency in the profiling of TCR specificities^158,159^. Leveraging on two well-characterized cohorts of *M. tuberculosis*-infected individuals, some of whom remained well (controllers) and others who progressed to active TB disease (progressors), Musvosvi et al. used scTCR-seq and the GLIPH2 algorithm to sequence *M. tuberculosis*-reactive T-cells (mostly CD4) and identify putative protective (enriched in controllers) or non-protective (enriched in progressors) TCR similarity groups^159^. Further, genome-wide antigen screening analysis allowed the identification of epitopes from two *M. tuberculosis* proteins, PE13 (Rv1195c) and CFP-10. These antigens were recognized by the TCR similarity group associated with controllers as putative vaccine targets. Interestingly, the majority of TCR similarity clusters were identified with equal frequencies in controllers and progressors^159^. This implies that *M. tuberculosis* expresses a number of antigens, which, despite inducing T-cells, have little impact on the outcome of TB infection. In addition, the authors showed that EspA (Rv3616c) is only recognized by T-cell clones in progressors^159^. Previously, it has been shown that EspA and CFP-10 are mutually dependent on each other for secretion in *M. tuberculosis*^39^. The opposite effect observed for TCR recognition of CFP10 and EspA, as well as the neutral effect of certain T-cell inducing antigens on outcome of TB infection, highlights the inadequacies of empirically selecting antigens and the need for systematic screening of T-cell specificities and TB outcome.

Immunopeptidomics is a recent advancement in mass-spectrometry-based approach for the precise identification of MHC-bound peptide epitopes^160^. This methodology identified 94 and 43 mycobacterial peptides presented by MHC class-II and MHC-I, respectively, in BCG-infected macrophages^161^. These peptides belonged to antigens predominantly expressed in infected macrophages, and a majority of them were membrane-associated and involved in lipid biosynthesis. Among these, three new antigens, GlfT2 (galactofuranosyl transferase, BCG_3870c (Rv3808c)), IniB (isoniazid inducible protein, Rv0341), and Fas (fatty acid synthetase, Rv2524c) were expressed in viral vectors and evaluated as boosters for BCG immunization^161^. A combination of these three antigens, when administered in a prime-boost regimen in intradermal BCG-immunized mice, showed significantly lower CFU counts in the lungs and spleen post-*M. tuberculosis* challenge compared to BCG-immunized mice. Immunopeptidomics has also been used to identify ligands bound to non-classical highly conserved HLA-E molecules from *M. tuberculosis*-infected human cells^162^. In this study, 28 HLA-E ligands were identified from 13 *M. tuberculosis* proteins. Among these, a peptide epitope was strongly and broadly recognized by CD8^+^ T-cells from donors with *M. tuberculosis* infection, latent infection, and healthy controls, suggesting it may have scope for vaccine strategy targeting donor-unrestricted T-cells.

Traditionally, TB vaccine antigen discovery approaches have focused on the identification of immunogenic protein antigens. However, mycobacterial lipid antigens are also attractive candidates for subunit TB vaccine development. Studies have shown that immunization of guinea pigs with formulations of mycobacterial lipid antigens imparts modest protection against *M. tuberculosis* challenge compared to unvaccinated guinea pigs^163^. Using a combination of protein and lipid antigens in multi-component subunit vaccines might hold promise for future TB vaccines. In a similar approach, immunization of humanized mice with a dual encapsulation of Ag85B and lipid antigen mycolic acid (MA) resulted in the activation of both protein and lipid antigen-specific T-cells^164^. Mycobacterial outer membrane vesicles (OMVs) containing membrane and cell wall components can be a source of mixed protein and lipid antigens and stimulate mixed humoral and cellular immune responses. OMVs have been evaluated in animal models, demonstrating their ability to impart protection against *M. tuberculosis* challenge^165–167^. However, heterogeneous size and low stability limit the use of OMVs as vaccines. OMV-coated nanoparticles have been shown to have improved stability and enhanced immuno-stimulatory properties^168^.

The majority of vaccine candidates proposed for TB vaccine development are focused on T-cell-mediated immunity. Earlier studies using *M. tuberculosis* infected cynomolgus macaques showed the presence of B-cell clusters surrounding granuloma that secrete *M. tuberculosis*-specific antibodies^169^. Whilst a functional role for humoral immunity in protection against *M. tuberculosis* remains poorly defined, a number of cohort studies have provided supporting evidence of the role of antibodies in the control of *M. tuberculosis* infection by mucosal immunity^170–179^. Studies have also demonstrated the role of monoclonal antibodies in imparting protection against TB in murine models^180^. Antibodies isolated from *M. tuberculosis-*exposed healthcare workers could confer moderate (~2.0- to 3.0-fold) protection against *M. tuberculosis* infection in aerosol mouse challenge model^173^. In addition, monoclonal antibodies against mycobacterial phosphate transporter subunit PstS1 (Rv0934) isolated during active TB infection showed inhibitory activity against intracellular growth of BCG and *M. tuberculosis* in THP-1 macrophages^9^. These observations suggest that protective antibody responses can be generated during active TB disease. Recently, a thorough analysis of IgG/IgA memory B-cell repertoire from an occupationally exposed, asymptomatic healthcare worker donor identified a monoclonal antibody targeting the cell membrane-associated virulence factor LpqH (Rv3763) as a promising candidate for future vaccine strategies^10^. Anti-LpqH human monoclonal antibody showed a reduction of *M. tuberculosis* counts in murine lungs when compared with a non-specific human isotype control^10^. These antibodies also displayed significant *M. tuberculosis* growth restriction in an ex vivo human whole blood mycobacterial growth inhibition assay^10^. Existing data strongly suggests that protective antibodies are directed against surface antigens of *M. tuberculosis*. Intravenous administration of antibody directed against surface antigen lipoarabinomannan (LAM) in intravenous *M. tuberculosis-*infected mice significantly reduces the bacterial burden in the lungs and spleens of infected animals and prolongs their survival^7^. Taken together, unbiased screening for potentially protective antibodies holds promise in identifying new targets for future TB vaccine development. Developing vaccines that target both cellular and humoral immunity may be an effective vaccination strategy against TB. However, there is a lack of data on epitopes that likely stimulate functional antibodies against *M. tuberculosis*, and cloning and characterization of protective human monoclonal antibodies can be a promising tool to identify antigens that induce such antibodies upon vaccination.

There has also been an increase in multi-epitope-based peptide vaccines for TB (TB MEVs) due to the recent advances in immunoinformatics and computational immunology. Immunoinformatics has expedited vaccine design by enabling in silico assessment of immunological properties of MEVs, including their ability to induce the proliferation of innate and adaptive immune cells and capacity to stimulate cytokine release from immune cells^181^. MEVs have demonstrated several advantages, such as appropriate safety profile, lower risk of adverse effects, and low manufacturing costs^182^. Helper T-lymphocyte (HTL) epitopes, cytotoxic T-lymphocyte (CTL) epitopes, and B-cell epitopes derived from several antigenic proteins of *M. tuberculosis* have been combined to create several TB MEVs. Multi-epitope vaccines that combine T-cell and B-cell epitopes can induce a broad range of immune responses. MEVs have been designed using epitopes from experimentally confirmed known antigenic proteins and antigens^183–187^. In silico tools are used to screen epitopes for their antigenicity, allergenicity and toxicity. These epitopes are fused with appropriate linkers and combined with TLR agonists as adjuvants. Subunit vaccine preparations of these MEVs can be purified by recombinant expression in *E. coli*^188^. The structural, physiochemical, and immunological attributes of the MEVs are validated using immunoinformatic tools, but most of these vaccines still need to be evaluated in vitro and in vivo for their immune and efficacy studies. ∼50 mRNA-based vaccines against SARS-CoV-2 were approved by the FDA in just three years. mRNA vaccines have the potential for quick and scalable vaccine production. Furthermore, mRNA vaccines address the safety concerns associated with the use of DNA vaccines that may cause insertional mutagenesis. There has been limited usage of RNA-based vaccination platforms in TB vaccine development. A recent study used immunoinformatics to design a multi-epitope mRNA vaccine based on predicted immunogenic epitopes from nine *M. tuberculosis* proteins. These proteins have been shown to modulate host immune responses by inducing epigenetic modifications^189^. BioNTech (Mainz, Germany) recently announced plans to test multi-antigenic mRNA-based TB vaccines BNT164a1 and BNT164b1 (ClinicalTrials.gov Identifier: NCT05547464) in phase I clinical trials for their safety and immunogenicity. Larsen et al. designed and evaluated a novel Venezuelan equine encephalitis (VEE) virus-based replicating RNA (repRNA) vaccine platform against *M. tuberculosis* infection in mice^190^. The ID91 repRNA is a multi-antigenic vaccine that harbors four *M. tuberculosis* antigens, EsxV (Rv3619, ESAT6-like protein), RpfD, PPE60 (Rv3478), and Ag85B in a VEE repRNA backbone. The efficacy of ID91 repRNA was compared to ID91, which was generated as a fusion protein formulated with a synthetic TLR4 agonist as a prophylactic vaccine against *M. tuberculosis* challenge in C57BL/6 mice. Immunization of mice with ID91 repRNA generated T-cell responses to a broader range of epitopes compared to ID91 protein subunit vaccine. Despite the better immune response, ID91 repRNA moderately reduced the lung bacterial burden compared to sham-immunized animals. In comparison, the heterologous RNA-prime protein-boost strategy was more effective in reducing the lung bacterial load^190^.

Taken together, advancements in antigen discovery approaches for vaccine candidates have exponentially increased the flux of antigens identified for designing vaccination strategies against *M. tuberculosis*. However, the majority of candidate antigens under clinical development were discovered decades ago. Small animal models are important preclinical models (i) for the evaluation of immunogenicity and protective efficacy of vaccine candidates and (ii) for prioritization of candidates into clinical trials. Although indispensable, no animal model fully recapitulates human TB disease. In the next section, we discuss recent advancements in preclinical evaluation models for TB vaccines.

## TB vaccine preclinical evaluation models

**Fig. 2:**
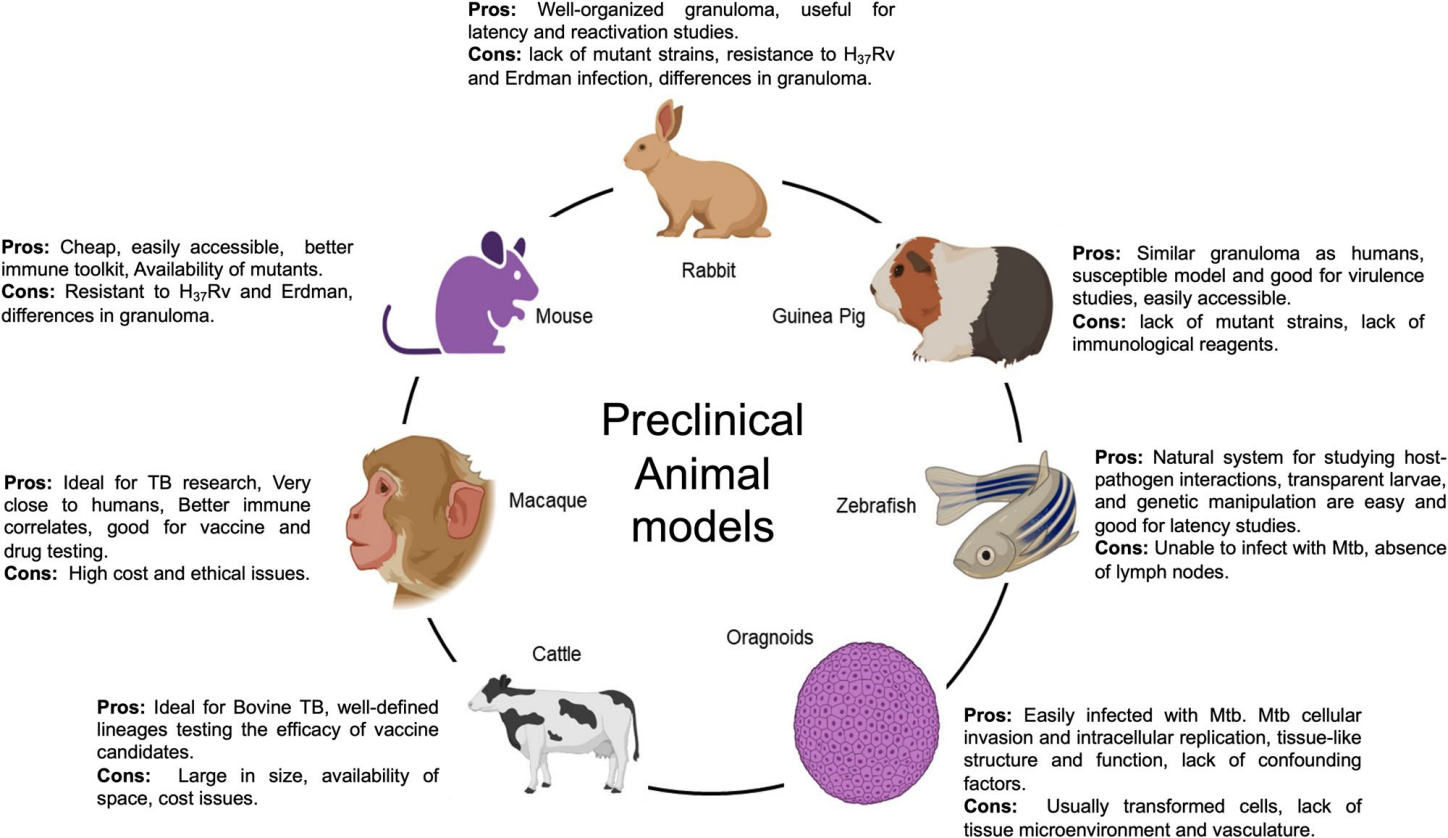
Animal models used in TB research. This figure highlights various animal models in TB vaccine development research and the advantages and disadvantages of each animal model. This figure was generated using BioRender.

Designing TB vaccine candidates with increased efficacy is facilitated by the advancements in antigen discovery approaches. Despite these advances, there are numerous challenges in developing a vaccine that is safe and imparts better protection than BCG in humans. These include (i) determining the entire antigenome of *M. tuberculosis*, (ii) choosing antigens or combinations that elicit optimal immunogenic responses, (iii) selecting antigens for epitope prediction, (iv) the necessity of combining T-cell and B-cell epitopes, (v) identifying appropriate vaccine correlates of protection and (vi) identifying preclinical evaluation models with predictive value for vaccine efficacy in humans. Animal models are valuable tools for studying immunogenicity, safety, and vaccine efficacy. Preclinical testing in animal models allows the screening of hundreds of vaccine candidates and helps prioritize a few for evaluation in human clinical trials. Various in vitro models and animal models, including mice, guinea pigs, rabbits, non-human primates and cattle have been used to study the efficacy of TB vaccines (Fig. 2). The advantages and disadvantages of conventional animal models for TB research have been extensively reviewed previously^191–193^. These animal models show considerable differences in their susceptibility to infection, the extent of organ involvement, and the type of immune response. For instance, immunization with MVA85A, a viral vectored vaccine constructed using modified vaccinia Ankara virus expressing antigen 85A, induced potent Th1 responses and imparted better protection than BCG in preclinical animal models but failed to deliver superior protection compared to BCG in Phase IIb clinical trial^194–196^. In addition, the route of vaccine administration also significantly impacts the outcome of the infection. For example, BCG is administered intradermally to the human population and shows variable efficacy (0–80%) against pulmonary TB^2,197^. A recent study showed that intravenous administration of BCG in non-human primates (*Macaca mulatta*) generated significantly enhanced CD4^+^ and CD8^+^ T-cell responses in blood, spleen, bronchoalveolar lavage, and lymph nodes compared to intradermal or aerosol administration of the vaccine. Further, intravenous immunization of BCG imparted protection in 90% of the macaques post *M. tuberculosis* challenge. Among these, 60% of the animals showed no detectable sign of infection^13^. The authors showed that intravenous immunization of non-human primates with BCG reduced the lung bacterial burden by greater than 1,00,000-fold compared to intradermal BCG immunization^13^. A higher proportion of mycobacteria-specific T-cells in airways, tissue-resident memory T-cells, and robust immunoglobulin IgM responses were identified as correlates of protection in macaques immunized intravenously with BCG^13,198^. Intravenous BCG immunization also protected simian immunodeficiency virus (SIV)-infected macaques from *M. tuberculosis* infection^199^. These studies demonstrate that BCG administration via the intravenous route is more immunogenic and efficacious than the conventional intradermally administered BCG. These observations suggest that intravenous administration of vaccines might boost the protective efficacy of vaccines that are under clinical development.

To eliminate TB, newer preclinical TB vaccine evaluation models are required to address key limitations of the existing animal models, which do not fully recapitulate features of human disease. In this review article, we discuss new in vitro, animal, and human challenge models that have been developed to complement the ongoing efforts in TB vaccine development and evaluation. These newer models may offer valuable insights into the quest to completely eradicate TB.

## Human challenge model

Human challenge (HC) models have been routinely used for various diseases such as malaria, influenza, dengue fever, and typhoid^200–203^. The advantages of the HC model include (i) a significant reduction in time and costs associated with preclinical assessment in animal models and (ii) identification of the correlates of protection. BCG is currently the strain of choice for infection in the HC model. BCG is safe for use in humans and has a 99.95% sequence similarity to *M. bovis*^204^. BCG can self-replicate and cause limited infection in immunocompetent animals and humans. Multiple approaches have been used to develop a less virulent self-replicating strain of *M*. *tuberculosis*. One strategy involves using *M. tuberculosis* auxotrophic strains that are attenuated for growth in macrophages and in vivo^205^. Another alternative approach entails using genetically modified strains expressing essential enzymes in the presence of either unnatural amino acid-based regulatory systems, regulated promoters, or regulatable self-kill switches. Self-kill switches are regulatory circuits that, in response to specific stimuli, can maintain essential gene expression or block the expression of toxins^206^. Without exogenous signals, these regulatory circuits block the expression of genes of interest or induce the expression of toxins^206–208^.

Angela et al. developed the first HC model, where intradermal BCG challenge was used as a surrogate for *M. tuberculosis* infection^209^. The rationale for choosing BCG as the infecting strain was based on the assumption that an effective vaccine candidate against *M. tuberculosis* will also reduce the BCG burden^209^. In this study, naive or BCG-vaccinated healthy volunteers were intradermally challenged with BCG, and the load of BCG was determined from skin biopsy specimens (challenge site) using culture and polymerase chain reaction (PCR). The authors showed that BCG persisted in the skin for more than four weeks and displayed a spectrum of protection among BCG-immunized groups. The majority of the cells in the blister fluid were neutrophils. PCR analysis of skin biopsy samples from vaccinated individuals showed a 10.0-fold reduction in challenge BCG counts compared to naïve individuals^210–212^. However, the culture data showed no difference in challenge load between the naive and BCG-vaccinated groups^209^. Earlier studies have shown that booster of BCG-immunized animals with MVA85A resulted in better protection in animals^213,214^. Harris et al. used BCG as the human challenge strain to evaluate the efficacy of MVA85A^215^. Unvaccinated and BCG-vaccinated individuals were immunized with MVA85A followed by intradermal challenge with BCG. It was observed that BCG challenge was well tolerated by individuals with an expected localized inflammatory reaction. The authors observed that prior BCG vaccination imparts some protection against subsequent BCG challenge. Further, a significant reduction in challenge strain was also detected in the BCG–MVA85A immunized group compared to the naive group. However, boosting BCG-vaccinated individuals with MVA85A resulted in no further reduction in the counts of challenge strain. Taken together, the results obtained are consistent with the outcomes of MVA85A phase 2b trials in BCG-vaccinated infants^6,215^. The authors showed that vaccination with BCG-MVA85A for seven days resulted in significantly higher ex vivo IFN-γ responses to Ag85A compared to BCG-naïve MVA85A vaccinated individuals, and this was consistent with previous reports in humans^215,216^. A lung-oriented controlled human infection model (CHIM) has been evaluated in South African volunteers using live BCG or sterile PPD bronchoscopically administered into the lungs establishing feasibility and safety of this human infection model with only mild adverse effects^217^. Recently, Wang et al. have described the construction of safe and effective *M. tuberculosis* strains for human challenge studies. This newly developed *M. tuberculosis* strain called the triple-kill-switch (TKS) strain has three kill switches tightly regulated via tetracyclines and trimethoprim. This TKS strain cannot replicate and grow in axenic cultures and mice in the absence of tetracyclines and trimethoprim. In contrast, under permissive conditions, the TKS strain displayed immunogenicity and antibiotic susceptibility similar to the parental strain^218^. These studies suggest that BCG and engineered *M. tuberculosis* strains might be considered for the human challenge model and provide a valuable tool for assessing vaccine-induced protection prior to clinical evaluation.

## Human PBMC-based in vitro models and organotypic cell culture models

In vitro three-dimensional cultures have also emerged as useful tools for studying tissue development, organogenesis, and stem cell behavior^219^. The hallmark of TB disease manifestation is granuloma formation^220^. To develop a three-dimensional culture model, PBMCs from healthy donors were incubated with partially purified PPD protein coated on cyanogen bromide-activated sepharose beads. In this model, the authors observed sequential recruitment of monocytes and lymphocytes around PPD-coated beads^221^. The cellular structure, thus formed, resembled the naturally occurring granulomas. The detailed analysis of these structures revealed that activated macrophages with large vesicles were the most prominent cell type. In addition, a few multinucleated giant macrophages and other non-immune cells, like epithelioid cells, were also recruited to the beads. Aliabbas et al. used PBMCs to identify and evaluate the T-cell activation by secretory antigens extracted from *M. tuberculosis* cultures at different stages of growth. PBMCs stimulated with CFPs isolated from the late logarithmic growth phase of *M. tuberculosis* culture exhibited better T-cell activity (measured by secretion of IFN-γ, TNF-α, and IL-12) when compared to the PBMCs induced with CFPs prepared from BCG strain^222^. Kapoor et al. developed an in vitro model of human TB granuloma using human primary leukocytes, where *M. tuberculosis* enters a dormancy-like state. In this model, PBMCs from healthy donors were incubated over a collagen-coated matrix in the presence of a low dose of *M. tuberculosis* for eight days, forming microscopic granulomas^223^. *M. tuberculosis* residing in these in vitro micro-granulomas exhibited characteristics of dormant mycobacteria, including (i) loss of acid-fastness, (ii) accumulation of lipid bodies, (iii) rifampicin resistance, and (iv) changes in gene expression. The salient features of this in vitro granuloma model included (i) lymphocyte aggregation around infected macrophages, (ii) formation of multinucleated giant cells, (iii) secretion of inflammatory cytokines by host cells, (iv) features of *M. tuberculosis* dormancy, and (v) reactivation of *M. tuberculosis* upon immune suppression by anti-TNFα monoclonal antibody treatment. In addition to human PBMC-based in vitro models, researchers have also generated an in vitro human lung tissue model using lung-tissue-specific epithelial cell lines, fibroblasts, primary human monocytes, and macrophages^224,225^. This organotypic culture model showed cellular characteristics of lung epithelium, such as even distribution of macrophages throughout the epithelium, extracellular matrix production, stratification of epithelium, and mucus secretion at air-liquid interphase^225,226^. Introduction of virulent *M. tuberculosis* H_37_Rv infected macrophages in this model resulted in granuloma-like clustering of macrophages at the site of infection resembling early events involved in TB granuloma formation^225^. Further, the authors also demonstrated that RD1 region and ESAT-6 secretion are necessary for the early-granuloma formation in this experimental lung tissue model reminiscent of in vivo studies^225^. This methodology has opened avenues to understanding host-pathogen interactions. Incorporating immune cells and vasculature to mimic the dynamic microenvironment of the lung airways can further improve the organoid models and enable us to study early immune responses post-infection.

## Genetically diverse mouse models

Host genetic variation is an important determinant of infection outcome. The genetic factors that govern the outcome of *M. tuberculosis* infection in humans are largely unknown^227^. Studies have shown that certain inbred mouse strains commonly used for vaccine efficacy testing are genetically more susceptible to *M. tuberculosis* infection compared to others. Based on the mean survival time (MST) upon infection with *M. tuberculosis*, mice strains have been categorized into two distinct groups. BALB/c, C57BL/6, and C57BL/10 are highly resistant to infection with MST of greater than 250 days. In comparison, DBA/2, C3H/HeJ, CBA, and 129/SvJ are highly susceptible to infection with MST of less than 114 days^228^. The genetic cross between C57BL/6 J (resistant) and C3HeB/FeJ (susceptible) was used to identify the *sst1* locus (susceptibility to tuberculosis locus 1) that regulates *M. tuberculosis* multiplication in lung tissues and disease progression^229^. The *sst1* locus encodes for an intracellular pathogen resistance 1 (*ipr1*) gene, which regulates innate immunity to *M. tuberculosis* infection. C3HeB/FeJ mice harbor a unique mutation in the *ipr1* allele, which leads to loss of expression of the *ipr1* gene, whereas other C3H strains like C3H/HeJ, C3H/HeSnJ, and C3H/HeOuJ encode for the wild-type protein^230^. Studies have shown that a mutated *ipr1* allele results in a defect in macrophage-mediated innate immune response upon infection with either *M. tuberculosis* or *Listeria monocytogenes*^230^. Ipr1 controls the multiplication of *M. tuberculosis* and *L. monocytogenes* by regulating apoptosis of infected cells. The lack of expression of Ipr1 results in necrosis and escape of *M. tuberculosis* from the phagolysosome^230^.

The resistance of mice to BCG is linked to the antimicrobial resistance gene *nramp1* (natural resistance-associated macrophage protein)^231^. *nramp1* encodes for an integral membrane transport protein, and the susceptibility allele harbors a non-conservative glycine-to-aspartic-acid substitution within the predicted transmembrane domain^232^. To determine whether *nramp1* contributes to resistance to *M. tuberculosis* infection, *nramp1*^*susc/susc*^ and *nramp1*^*res/res*^ mice were infected with *M. tuberculosis* via intravenous or aerosol route. Both mice strains were susceptible to infection with *M*. *tuberculosis* at comparable levels^231,233^. These observations demonstrated that *nramp1* is not involved in resistance to *M. tuberculosis* infection. Inbred mice have been widely used for TB research, but a significant limitation of this model is that they fail to mimic the genetic heterogeneity seen in humans^234,235^. Optimally utilizing the existing genetic diversity within the mouse species can enhance its relevance and mirror essential pathological features resembling those in humans. The diversity outbred (DO) model is derived from partially inbred mice strains and is maintained by randomized crossing to create a diverse experimental population that reduces erroneous interpretations due to strain-specific effects^236^. DO mice have been used as a tool to study (i) susceptibility to *M. tuberculosis* infection, (ii) variability in inflammatory responses in a heterogeneous population, and (iii) identify immune correlates of TB disease^237,238^. Ahmed et al. performed a detailed analysis of the global blood transcriptomics data obtained from the lungs of DO mice and primates and found that the expression of 13 genes (*hist2h2bb*, *pram1*, *icam1*, *kctd12*, *nub1*, *h2afj*, *folr2*, *h3f3a*, *dnttip1*, *ltbr*, *mrpl23*, *mfsd14b*, and *scarf1*) correlated well with active disease progression across species^239^. Among these, Scarf1 plays a crucial role in regulating innate immunity, immunopathogenesis, and tissue destruction, and is involved in apoptosis. In agreement, *scarf1* knockout mice showed reduced disease compared to wild-type mice following *M. tuberculosis* infection^239^. The expression of *sept4* encoding a nucleotide-binding protein involved in cytoskeleton rearrangements was increased in humans and macaques following infection with *M. tuberculosis*. However, in mice tissues, *sept4* expression remained unchanged during infection^239^. Therefore, these observations suggest Sept4 might function differentially in mice when compared to primates and humans. Serping1 (serpin family G member 1), a highly glycosylated plasma protein, negatively regulates the complement system. Despite increased expression upon infection with *M. tuberculosis*, the extent of disease progression was similar in wild-type and *serping1*^−/−^ mice at different stages of infection^239^. Tap1 is important for antigen presentation to CD8^+^ T-cells and was upregulated upon infection with *M. tuberculosis*. In agreement, it was reported that *tap*^*-/-*^ mice are unable to control bacterial burden during acute and chronic stages of disease^239^.

DO mice are well suited for studying vaccine efficacy due to their genotypic and phenotypic heterogeneity. DO mice challenged with low doses of *M. tuberculosis* display a range of disease susceptibility and progression. Further, immunization of DO mice with BCG is able to impart protection, resulting in increased survival and reduction in organ bacterial load^240^. A recent study showed that intravenous immunization of DO mice with BCG imparted moderately better protection compared to intradermally immunized DO mice^241^. In addition to the DO model, the Collaborative Cross (CC) mouse model has also emerged as a useful model to understand the impact of host genetic background on protection imparted by vaccination^242,243^. The CC mouse model was developed using eight diverse founder strains and recapitulated the heterogeneity of the human genome^244–247^. The CC mouse panels have been used to evaluate the protection imparted by subcutaneous immunization of BCG against *M. tuberculosis* challenge^242^. To investigate the impact of the host genetic background on the protective efficacy of BCG against *M. tuberculosis* challenge, 24 distinct CC strains were subcutaneously vaccinated with BCG. The authors showed that immunization with BCG protects half (13 out of 24 CC strains) of the mouse strains, suggesting a high degree of variability among the CC mouse strains. These observations suggest that host genetics has a major influence on BCG-induced immunity against *M. tuberculosis* infection^242^. Therefore, identifying the factors that regulate immunity will improve our understanding of host-pathogen interactions and help design more effective TB vaccines.

Another factor that limits the use of traditional mouse models of TB disease is the challenge dose of *M. tuberculosis*. The infection dose in “conventional” mice models typically varies between ~50–100 bacterial colony-forming units (CFUs), resulting in uniformly high lung bacterial burdens, which are associated with progressive inflammatory disease and poorly organized granulomas^248^. On the contrary, only ~1–3 bacilli are sufficient to establish disease in humans in most cases^249,250^. To mimic human-like infection conditions, Plumlee et al. developed an ultra-low dose (ULD) mice infection model^248^. The authors showed that mice infected with ~ 1–3 CFUs of *M. tuberculosis* exhibited highly heterogeneous bacterial burdens and well-circumscribed granulomas that shared features with human granulomas. This study also demonstrated that transcriptional signatures derived from whole-blood of ULD or conventional dose mice model correlated well with lung bacterial burden and predicted *M. tuberculosis* infection outcomes across species, including the risk of progression to active TB in humans^248^. In another study, the ULD mouse model was used to assess the protective efficacy of the attenuated Δ*lprG* mutant strain of *M. tuberculosis*. The authors showed that immunization of mice with Δ*lprG* strain imparted significantly better protection against aerosol *M. tuberculosis* challenge than immunization with BCG^251^. Immunization of mice with Δ*lprG* reduced lung bacillary load by ∼20.0-fold and 5.0-fold, respectively, compared to the naive and BCG-immunized group^251^.

## Humanized mice model

Humanized mice are immune-compromised mice engrafted with human cells or tissues. These mice are deficient in mature T and B lymphocytes and harbor a mutation in the *prkdc* locus, which is involved in the rearrangement of T-cell Receptor and Immunoglobulin genes^252^. The major disadvantage of the humanized mice model is graft rejection. The major advantage associated with humanized mice is that these mice mimic the essential characteristics of the human immune response to *M. tuberculosis* infection. Rottenberg et al. showed that infection of humanized mice with *M. tuberculosis* resulted in human-like granulomas^253^. Subsequently, this model has been used to study immune responses and evaluate the efficacy of various drug regimens against *M. tuberculosis*. Calderon et al. generated humanized bone marrow-liver-thymus (BLT) mice by transplanting human fetal liver and thymus supplemented with CD34^+^ fetal liver cells in immunodeficient NOD/SCID/IL-2Rγ^−/−^ (NSG) mice^254^. The authors observed that these mice generated functional leukocytes, T-cells, natural killer cells, and monocyte/macrophages at 12 weeks post-engraftment^254^. These mice were infected intranasally with *M. tuberculosis*, and the infection resulted in organized granulomatous lesions, caseous necrosis, bronchial obstruction, and crystallization of cholesterol deposits at the site of infection, indicating pathology similar to human disease. Moreover, human leukocytes showed migration to the spleen, lung, and liver, and functional CD4^+^ and CD8^+^ T-cell subsets were observed in these tissues^254^. Lee et al. also generated BLT mice by transplantation of human fetal liver, thymus, and hematopoietic stem cells in severely immunodeficient NSG mice^255^. The authors showed that BCG-infected NSG mice generated a higher number of IFN-γ producing cells. However, despite increased numbers of IFN-γ producing cells, the transgenic mice were susceptible to infection with BCG^255^. Angelo et al. used HuMurine’s humanized NOG (Hu-M^TM^) mice to assess the efficacy of standard BCG vaccine or a vaccine containing CpG-C adjuvant, an ESAT-6-based liposomal formulation^256^. The authors demonstrated that the immune responses generated by the humanized mice were comparable to those observed in C57BL/6 mice and guinea pigs. The immunization of humanized mice resulted in the induction of human CD4^+^ and CD8^+^ T-cells and the expression of various cytokines such as IFN-γ, IL-2, and TNF-α, which are known to confer better protection against *M. tuberculosis*. Collectively, results obtained with humanized mice is similar to the data observed in C57BL/6 mice and guinea pigs, suggesting immunization with vaccine can induce T-cell response in humanized mice^256^. Many mycobacterial cell wall lipids are known to be presented by non-polymorphic human CD1 molecules, resulting in the stimulation of unconventional T-cell subsets^257^. Zhao et al., have developed a transgenic mice model that expresses proteins such as group 1 CD1 molecules (hCD1Tg) and a CD1b-restricted, mycolic-acid specific TCR (DN1Tg) that are necessary for the presentation of lipid antigens to T-cells^258^. The authors showed that aerosol *M. tuberculosis* infection of these mice resulted in the generation of CD-1-restricted *M. tuberculosis* lipid-specific activated polyfunctional T-cells. In addition, the adoptive transfer of DN1 T-cells in hCD1Tg/Rag^−/−^ mice prior to the *M. tuberculosis* challenge resulted in reduced viable bacterial counts by ~ 5.0, 10.0, and 10.0-fold in lungs, spleens, and liver, respectively, compared to mice that received no DN1 T-cell transfer^258^. Immunization of humanized mice with an adenovirus-based AdHu5Ag85A resulted in the generation of CD4^+^ T-cell response^259^. Further, the polyfunctional properties of CD4^+^ T-cells from humanized mice were comparable to the response observed in participants enrolled in a Phase I clinical study^259,260^. Taken together, the humanized mice model offers a promising surrogate model for assessing the effectiveness of potential vaccines before proceeding to clinical trials.

## Zebrafish model of infection

*M. marinum* infection of zebrafish is emerging as a model to study TB infections. Mycobacterial infection in humans and zebrafish shows similar latent infection features and spontaneous disease reactivation^261^. The zebrafish model of infection offers several advantages in laboratory settings. Firstly, adult zebrafish and embryos can be easily infected. Secondly, the transparency of zebrafish embryos allows for the use of advanced optical techniques, leading to more profound insights into the infection dynamics. Thirdly, genetic manipulations in zebrafish are relatively easy, and infection with *M. marinum* results in macrophage recruitment. Zebrafish and humans share a common locus for pro- and anti-inflammatory cytokine regulation^262^. Zebrafish have been used as a model for TB vaccine evaluation, and immunization of zebrafish with BCG resulted in improved survival rate compared to saline control against both low and high doses of *M. marinum* infection^263,264^. In another study, immunization of zebrafish with an attenuated *M. marinum* strain (L1D) harboring a deletion in the PE/PGRS family of proteins (MAG 24-1) was able to impart protection against challenge with the virulent *M. marinum* OSU-214 strain. The authors observed a significantly improved fish survival rate compared to vaccination with culture filtrate proteins (CFP) or heat-killed OSU-214 strain or control group^265,266^. Intramuscular immunization of zebrafish with DNA vaccines encoding Ag85C, CFP-10, and ESAT-6 (pCMV-Ag85B/CFP-10/ESAT-6) resulted in fewer granulomas and reduced mycobacterial dissemination following low dose *M. marinum* infection^263^. In addition, immunization with DNA vaccines resulted in a higher survival rate of zebrafish infected with high doses of *M. marinum* when compared to sham immunized control^263^. The observed protection was dependent on cell-mediated adaptive immune response as immunization with pCMV-Ag85B/CFP-10/ESAT-6 was unable to enhance the survival rate of Rag1 (recombination activation protein 1) deficient zebrafish that lack functional T- and B- lymphocytes^263^. In another study, immunization of zebrafish with a DNA vaccine overexpressing Ag85B, ESAT-6, and RpfE enhanced the protective efficacy imparted by BCG and the survival rate^264^. To identify new antigen candidates using the zebrafish model, 15 *M. marinum* antigens that showed differential expression during the mycobacterial infection cycle were assessed as DNA vaccine candidates in both low and high doses of *M. marinum* infection^261^. In a low-dose infection model, immunization of zebrafish with DNA vaccines encoding RpfE, PE5_1, PE31, and Cdh (*M. tuberculosis* homologs include RpfE/ Rv2450c, PE15/Rv1386, PE13, and Rv2289, respectively) reduced the bacterial burden by 50-88% compared to zebrafish immunized with the plasmid vector^261^. However, only the DNA vaccine expressing RpfE significantly improved the survival rate of zebrafishes compared to controls in a high-dose infection model^261^. In agreement, subcutaneous immunization of mice with RpfE protein has been shown to confer a high level of protection against *M. tuberculosis* challenge^95^. The authors observed 100.0-fold and 10.0-fold CFU reduction in vaccinated animals’ lungs and spleens, respectively, compared to sham-immunized mice. The same 15 differentially expressed antigens were also used to identify post-exposure protective antigens in a zebrafish dexamethasone-induced reactivation model following *M. marinum* infection^267^. The authors showed that RpfB and MMAR_4207 protect TB reactivation when evaluated as a post-exposure DNA vaccine in an immunosuppression-based reactivation model of zebrafish. Among Rpf homologs, *M. tuberculosis* RpfB has emerged as an immunogenic and promising vaccine candidate antigen^12,96,97,99^. Taken together, zebrafish has emerged as a useful model to (i) study functions of the innate and adaptive immune response, (ii) study latency, dormancy, and disease reactivation, and (iii) for preclinical evaluation of potential vaccine candidates^268^.

## Conclusion

New and more effective vaccines are urgently needed to eradicate TB. In view of the fact that only a small portion of the *M. tuberculosis* antigenome has been explored for designing better TB vaccination strategies, innovation in antigen discovery is a crucial aspect of TB research. BCG, although prevents disseminated TB in children, its major drawback is the variable efficacy against pulmonary TB in adults and limited effect on TB transmission^269^. Better protective vaccines are needed to complement or substitute BCG. Identification of antigens that induce protective responses that correlate with natural protection to *M. tuberculosis* infection, disease or disease recurrence in humans is critical for vaccine design. New protective antigens are needed, also in view of the failure of MVA85A in BCG-vaccinated infants^196^. To date, a range of approaches have been used to identify many promising vaccine-candidate antigens and epitopes. Traditional methods of identification of immunogenic proteins in the pre-genomic era involved isolation and biochemical characterization of *M. tuberculosis* proteins expressed during in vitro culture. Advancements in antigen discovery approaches have helped improve our understanding of the *M. tuberculosis* antigenome and increased the flux of candidate vaccines in preclinical testing. Over the last two decades, many potentially protective *M. tuberculosis* antigens have been discovered using genome-wide approaches and bioinformatics tools. However, only a handful of antigens have been evaluated as candidate vaccine targets in preclinical and clinical settings with variable success in protecting against TB compared to BCG. In addition to secretory antigens, dormancy-associated antigens and resuscitation-promoting factors have emerged as promising targets for development of TB vaccination strategies especially considering the inability of BCG to induce T-cell responses against dormancy antigens^270,271^. One factor that has hindered the identification of protective antigens is the complexity of the immune response against *M. tuberculosis*. Immunological readouts focused on IFN-γ production may not comprehensively capture the *M. tuberculosis* antigenome, and identification of antigens that trigger unconventional T-cell responses may be necessary for designing TB vaccines equipped to trigger a wider range of immune responses against *M. tuberculosis*^272^. While crucial role of cell-mediated immunity in protection against TB has been established, recent identification of naturally occurring antibodies that are protective against *M. tuberculosis* infection in humans suggests that antibody-mediated immunity may also play an important role in designing future vaccines against TB^180^. To propel vaccine candidates in TB vaccine pipeline, it is crucial to evaluate their immunogenicity and protective efficacy in animal models of relevance to human TB disease. Since a single animal model is unable to fully recapitulate the complexity of human disease, there is a need to evaluate the effectiveness of TB vaccine candidates in multiple preclinical models to predict their efficacy in humans. The existing animal models, such as mice, guinea pigs, and NHP, have been useful to evaluate the safety, immunogenicity, and protective efficacy of potential vaccine candidates. These models are helpful, but more preclinical animal models and in vitro models that can recapitulate discerning features of human TB disease are required. The recently reported diversity outbred (DO), collaborative cross (CC) and ultra-low dose (ULD) mouse models offer a better choice over conventional models for investigating the influence of host genetics on disease outcome and protection imparted by BCG. The humanized mice and organoid models provide an advantage in studying human-like granuloma and immune response. In addition, the human challenge model would facilitate the TB vaccine development pipeline and would aid in 1) the selection of vaccine candidates for efficacy trials, 2) optimizing dose and route of immunization, and 3) the identification of immune correlates of protection. Although there have been significant advancements in TB vaccine research there are still challenges in designing effective TB vaccines. These include an incomplete understanding of the pathogenicity of *M. tuberculosis*, lack of correlates of protection, and absence of an appropriate animal model to evaluate vaccines. Despite these challenges, immunization of non-human primates with RhCMV/TB and IV BCG has shown sterilizing immunity in a proportion of vaccinated animals and has renewed enthusiasm for TB vaccine research^11,13^. Intravenous administration of BCG was found to induce superior protection than intradermal or aerosol delivery, as observed by reduction in mycobacterial growth, pathology and granuloma formation in various tissues, with no detectable *M. tuberculosis* infection in six out of ten macaques receiving IV BCG^13^. The T-cell response induced by intravenous BCG immunization was mostly of Th1 type with some contribution of Th17 type. This unprecedented protection against *M. tuberculosis* challenge conferred by IV BCG administration suggests that the efficacy of BCG itself can be improved by appropriate route of administration and argues against the notion that BCG lacks antigens required for eliciting protective immune response and that protective antigens are critical for designing effective vaccination strategies against TB. However, transient splenomegaly was observed following intravenous administration of BCG in NHPs and it remains to be seen how this may pose a hindrance to clinical studies in humans. Presently, given the success of two vaccine candidates in NHP models and promising proof-of-concept phase IIb efficacy trial of M72/AS01E in HIV-negative adults with LTBI, it is imperative to prioritize the identification of correlates of protection relevant to the human population that can be further validated in clinical trials.

## References

[1] Trunz BB, Fine P, Dye C (2006). Effect of BCG vaccination on childhood tuberculous meningitis and miliary tuberculosis worldwide: a meta-analysis and assessment of cost-effectiveness. Lancet.

[2] Fine PE (1995). Variation in protection by BCG: implications of and for heterologous immunity. Lancet.

[3] Colditz GA (1995). The efficacy of bacillus Calmette-Guerin vaccination of newborns and infants in the prevention of tuberculosis: meta-analyses of the published literature. Pediatrics.

[4] Rodrigues LC, Diwan VK, Wheeler JG (1993). Protective effect of BCG against tuberculous meningitis and miliary tuberculosis: a meta-analysis. Int J. Epidemiol..

[5] Louise R, Skjot V, Agger EM, Andersen P (2001). Antigen discovery and tuberculosis vaccine development in the post-genomic era. Scand. J. Infect. Dis..

[6] Tameris MD (2013). Safety and efficacy of MVA85A, a new tuberculosis vaccine, in infants previously vaccinated with BCG: a randomised, placebo-controlled phase 2b trial. Lancet.

[7] Hamasur B (2004). A mycobacterial lipoarabinomannan specific monoclonal antibody and its F(ab’) fragment prolong survival of mice infected with Mycobacterium tuberculosis. Clin. Exp. Immunol..

[8] Teitelbaum R (1998). A mAb recognizing a surface antigen of Mycobacterium tuberculosis enhances host survival. Proc. Natl Acad. Sci. USA.

[9] Watson A (2021). Human antibodies targeting a Mycobacterium transporter protein mediate protection against tuberculosis. Nat. Commun..

[10] Krishnananthasivam S (2023). An anti-LpqH human monoclonal antibody from an asymptomatic individual mediates protection against Mycobacterium tuberculosis. NPJ Vaccines.

[11] Hansen SG (2018). Prevention of tuberculosis in rhesus macaques by a cytomegalovirus-based vaccine. Nat. Med.

[12] Leung-Theung-Long S (2018). A multi-antigenic MVA vaccine increases efficacy of combination chemotherapy against Mycobacterium tuberculosis. PLoS One.

[13] Darrah PA (2020). Prevention of tuberculosis in macaques after intravenous BCG immunization. Nature.

[14] Baldwin SL (1998). Evaluation of new vaccines in the mouse and guinea pig model of tuberculosis. Infect. Immun..

[15] Andersen P, Askgaard D, Ljungqvist L, Bentzon MW, Heron I (1991). T-cell proliferative response to antigens secreted by Mycobacterium tuberculosis. Infect. Immun..

[16] Orme IM (1988). Induction of nonspecific acquired resistance and delayed-type hypersensitivity, but not specific acquired resistance in mice inoculated with killed mycobacterial vaccines. Infect. Immun..

[17] Orme IM, Andersen P, Boom WH (1993). T cell response to Mycobacterium tuberculosis. J. Infect. Dis..

[18] Boesen H, Jensen BN, Wilcke T, Andersen P (1995). Human T-cell responses to secreted antigen fractions of Mycobacterium tuberculosis. Infect. Immun..

[19] Roberts AD (1995). Characteristics of protective immunity engendered by vaccination of mice with purified culture filtrate protein antigens of Mycobacterium tuberculosis. Immunology.

[20] Young DB, Kaufmann SH, Hermans PW, Thole JE (1992). Mycobacterial protein antigens: a compilation. Mol. Microbiol.

[21] Andersen P (1994). Effective vaccination of mice against Mycobacterium tuberculosis infection with a soluble mixture of secreted mycobacterial proteins. Infect. Immun..

[22] Pal PG, Horwitz MA (1992). Immunization with extracellular proteins of Mycobacterium tuberculosis induces cell-mediated immune responses and substantial protective immunity in a guinea pig model of pulmonary tuberculosis. Infect. Immun..

[23] Nagai S, Wiker HG, Harboe M, Kinomoto M (1991). Isolation and partial characterization of major protein antigens in the culture fluid of Mycobacterium tuberculosis. Infect. Immun..

[24] Andersen P (1992). Identification of immunodominant antigens during infection with Mycobacterium tuberculosis. Scand. J. Immunol..

[25] Horwitz MA, Lee BW, Dillon BJ, Harth G (1995). Protective immunity against tuberculosis induced by vaccination with major extracellular proteins of Mycobacterium tuberculosis. Proc. Natl Acad. Sci. USA.

[26] Sinha RK, Verma I, Khuller GK (1997). Immunobiological properties of a 30 kDa secretory protein of Mycobacterium tuberculosis H37Ra. Vaccine.

[27] Andersen P, Askgaard D, Ljungqvist L, Bennedsen J, Heron I (1991). Proteins released from Mycobacterium tuberculosis during growth. Infect. Immun..

[28] Abou-Zeid C (1988). Characterization of fibronectin-binding antigens released by Mycobacterium tuberculosis and Mycobacterium bovis BCG. Infect. Immun..

[29] Barnes PF (1992). Immunoreactivity of a 10-kDa antigen of Mycobacterium tuberculosis. J. Immunol..

[30] Haslov K (1995). Guinea pig cellular immune responses to proteins secreted by Mycobacterium tuberculosis. Infect. Immun..

[31] Launois P (1994). T-cell-epitope mapping of the major secreted mycobacterial antigen Ag85A in tuberculosis and leprosy. Infect. Immun..

[32] Roche PW (1994). Differential T cell responses to mycobacteria-secreted proteins distinguish vaccination with bacille Calmette-Guerin from infection with Mycobacterium tuberculosis. J. Infect. Dis..

[33] Daniel TM, Ferguson LE (1970). Purification and Characterization Of Two Proteins From Culture Filtrates of Mycobacterium tuberculosis H(37)Ra Strain. Infect. Immun..

[34] Fukui Y, Hirai T, Uchida T, Yoneda M (1965). Extracellular proteins of tubercle bacilli. IV. Alpha and beta antigens as major extracellular protein products and as cellular components of a strain (H37Rv) of Mycobacterium tuberculosis. Biken J..

[35] Wiker HG, Harboe M (1992). The antigen 85 complex: a major secretion product of Mycobacterium tuberculosis. Microbiol. Rev..

[36] Belisle JT (1997). Role of the major antigen of Mycobacterium tuberculosis in cell wall biogenesis. Science.

[37] Andersen P, Andersen AB, Sorensen AL, Nagai S (1995). Recall of long-lived immunity to Mycobacterium tuberculosis infection in mice. J. Immunol..

[38] Berthet FX, Rasmussen PB, Rosenkrands I, Andersen P, Gicquel B (1998). A Mycobacterium tuberculosis operon encoding ESAT-6 and a novel low-molecular-mass culture filtrate protein (CFP-10). Microbiology.

[39] Fortune SM (2005). Mutually dependent secretion of proteins required for mycobacterial virulence. Proc. Natl Acad. Sci. USA.

[40] Parkash O, Singh BP, Pai M (2009). Regions of differences encoded antigens as targets for immunodiagnosis of tuberculosis in humans. Scand. J. Immunol..

[41] Bottai D (2015). Increased protective efficacy of recombinant BCG strains expressing virulence-neutral proteins of the ESX-1 secretion system. Vaccine.

[42] Pym AS (2003). Recombinant BCG exporting ESAT-6 confers enhanced protection against tuberculosis. Nat. Med..

[43] Heijmenberg I (2021). ESX-5-targeted export of ESAT-6 in BCG combines enhanced immunogenicity & efficacy against murine tuberculosis with low virulence and reduced persistence. Vaccine.

[44] Andersen AB, Yuan ZL, Haslov K, Vergmann B, Bennedsen J (1986). Interspecies reactivity of five monoclonal antibodies to Mycobacterium tuberculosis as examined by immunoblotting and enzyme-linked immunosorbent assay. J. Clin. Microbiol..

[45] Jungblut PR (1999). Comparative proteome analysis of Mycobacterium tuberculosis and Mycobacterium bovis BCG strains: towards functional genomics of microbial pathogens. Mol. Microbiol..

[46] Coler RN (1998). Molecular cloning and immunologic reactivity of a novel low molecular mass antigen of Mycobacterium tuberculosis. J. Immunol..

[47] Coler RN (2001). Vaccination with the T cell antigen Mtb 8.4 protects against challenge with Mycobacterium tuberculosis. J. Immunol..

[48] Covert BA, Spencer JS, Orme IM, Belisle JT (2001). The application of proteomics in defining the T cell antigens of Mycobacterium tuberculosis. Proteomics.

[49] Ernst JD (2012). The immunological life cycle of tuberculosis. Nat. Rev. Immunol..

[50] Wayne LG (1960). The bacteriology of respected tuberculous pulmonary lesions. 2. Observations on bacilli which are stainable but which cannot be cultured. Am. Rev. Respir. Dis..

[51] Rosenkrands I (2002). Hypoxic response of Mycobacterium tuberculosis studied by metabolic labeling and proteome analysis of cellular and extracellular proteins. J. Bacteriol..

[52] Wayne LG, Hayes LG (1996). An in vitro model for sequential study of shiftdown of Mycobacterium tuberculosis through two stages of nonreplicating persistence. Infect. Immun..

[53] Sherman DR (2001). Regulation of the Mycobacterium tuberculosis hypoxic response gene encoding alpha -crystallin. Proc. Natl Acad. Sci. USA.

[54] Yuan Y, Crane DD, Barry CE (1996). Stationary phase-associated protein expression in Mycobacterium tuberculosis: function of the mycobacterial alpha-crystallin homolog. J. Bacteriol..

[55] Cunningham AF, Spreadbury CL (1998). Mycobacterial stationary phase induced by low oxygen tension: cell wall thickening and localization of the 16-kilodalton alpha-crystallin homolog. J. Bacteriol..

[56] Yuan Y (1998). The 16-kDa alpha-crystallin (Acr) protein of Mycobacterium tuberculosis is required for growth in macrophages. Proc. Natl Acad. Sci. USA.

[57] Caccamo N (2002). Identification of epitopes of Mycobacterium tuberculosis 16-kDa protein recognized by human leukocyte antigen-A*0201 CD8(+) T lymphocytes. J. Infect. Dis..

[58] Wilkinson RJ (1998). Human T- and B-cell reactivity to the 16kDa alpha-crystallin protein of Mycobacterium tuberculosis. Scand. J. Immunol..

[59] Vordermeier HM (1993). Recognition of peptide epitopes of the 16,000 MW antigen of Mycobacterium tuberculosis by murine T cells. Immunology.

[60] Friscia G (1995). Human T cell responses to peptide epitopes of the 16-kD antigen in tuberculosis. Clin. Exp. Immunol..

[61] Demissie A (2006). Recognition of stage-specific mycobacterial antigens differentiates between acute and latent infections with Mycobacterium tuberculosis. Clin. Vaccin. Immunol..

[62] Shi C (2010). Enhanced protection against tuberculosis by vaccination with recombinant BCG over-expressing HspX protein. Vaccine.

[63] Dey B (2011). Latency antigen alpha-crystallin based vaccination imparts a robust protection against TB by modulating the dynamics of pulmonary cytokines. PLoS One.

[64] Spratt JM, Britton WJ, Triccas JA (2010). In vivo persistence and protective efficacy of the bacille Calmette Guerin vaccine overexpressing the HspX latency antigen. Bioeng. Bugs.

[65] Roupie V (2007). Immunogenicity of eight dormancy regulon-encoded proteins of Mycobacterium tuberculosis in DNA-vaccinated and tuberculosis-infected mice. Infect. Immun..

[66] Khera A (2005). Elicitation of efficient, protective immune responses by using DNA vaccines against tuberculosis. Vaccine.

[67] Chauhan P, Jain R, Dey B, Tyagi AK (2013). Adjunctive immunotherapy with alpha-crystallin based DNA vaccination reduces Tuberculosis chemotherapy period in chronically infected mice. Sci. Rep..

[68] Dey B (2011). A booster vaccine expressing a latency-associated antigen augments BCG-induced immunity and confers enhanced protection against tuberculosis. PLoS One.

[69] Taylor JL (2012). HspX-mediated protection against tuberculosis depends on its chaperoning of a mycobacterial molecule. Immunol. Cell Biol..

[70] Li Q (2011). Immunogenicity and protective efficacy of a fusion protein vaccine consisting of antigen Ag85B and HspX against Mycobacterium tuberculosis infection in mice. Scand. J. Immunol..

[71] Nangpal P, Bahal RK, Tyagi AK (2017). Boosting with recombinant MVA expressing M. tuberculosis alpha-crystallin antigen augments the protection imparted by BCG against tuberculosis in guinea pigs. Sci. Rep..

[72] Chen L (2010). The development and preliminary evaluation of a new Mycobacterium tuberculosis vaccine comprising Ag85b, HspX and CFP-10:ESAT-6 fusion protein with CpG DNA and aluminum hydroxide adjuvants. FEMS Immunol. Med. Microbiol..

[73] Betts JC, Lukey PT, Robb LC, McAdam RA, Duncan K (2002). Evaluation of a nutrient starvation model of Mycobacterium tuberculosis persistence by gene and protein expression profiling. Mol. Microbiol.

[74] Yihao D, Hongyun H, Maodan T (2015). Latency-associated protein Rv2660c of Mycobacterium tuberculosis augments expression of proinflammatory cytokines in human macrophages by interacting with TLR2. Infect. Dis..

[75] Schnappinger D (2003). Transcriptional Adaptation of Mycobacterium tuberculosis within Macrophages: Insights into the Phagosomal Environment. J. Exp. Med..

[76] Aagaard C (2011). A multistage tuberculosis vaccine that confers efficient protection before and after exposure. Nat. Med..

[77] Lin PL (2012). The multistage vaccine H56 boosts the effects of BCG to protect cynomolgus macaques against active tuberculosis and reactivation of latent Mycobacterium tuberculosis infection. J. Clin. Invest.

[78] Bertholet S (2008). Identification of human T cell antigens for the development of vaccines against Mycobacterium tuberculosis. J. Immunol..

[79] Coler RN (2018). The TLR-4 agonist adjuvant, GLA-SE, improves magnitude and quality of immune responses elicited by the ID93 tuberculosis vaccine: first-in-human trial. NPJ Vaccines.

[80] Voskuil MI (2003). Inhibition of respiration by nitric oxide induces a Mycobacterium tuberculosis dormancy program. J. Exp. Med..

[81] Bivas-Benita M (2009). Pulmonary delivery of DNA encoding Mycobacterium tuberculosis latency antigen Rv1733c associated to PLGA-PEI nanoparticles enhances T cell responses in a DNA prime/protein boost vaccination regimen in mice. Vaccine.

[82] Black GF (2009). Immunogenicity of novel DosR regulon-encoded candidate antigens of Mycobacterium tuberculosis in three high-burden populations in Africa. Clin. Vaccin. Immunol..

[83] Coppola M (2015). Synthetic long peptide derived from mycobacterium tuberculosis latency antigen Rv1733c protects against tuberculosis. Clin. Vaccin. Immunol..

[84] Reece ST (2011). Improved long-term protection against Mycobacterium tuberculosis Beijing/W in mice after intra-dermal inoculation of recombinant BCG expressing latency associated antigens. Vaccine.

[85] Kwon KW (2017). Novel vaccine potential of Rv3131, a DosR regulon-encoded putative nitroreductase, against hyper-virulent Mycobacterium tuberculosis strain K. Sci. Rep..

[86] Leyten EM (2006). Human T-cell responses to 25 novel antigens encoded by genes of the dormancy regulon of Mycobacterium tuberculosis. Microbes Infect..

[87] Commandeur S (2011). Double- and monofunctional CD4(+) and CD8(+) T-cell responses to Mycobacterium tuberculosis DosR antigens and peptides in long-term latently infected individuals. Eur. J. Immunol..

[88] Goletti D (2010). Response to Rv2628 latency antigen associates with cured tuberculosis and remote infection. Eur. Respir. J..

[89] Jung ID (2014). Enhancement of tumor-specific T cell-mediated immunity in dendritic cell-based vaccines by Mycobacterium tuberculosis heat shock protein X. J. Immunol..

[90] Bhatt P, Sharma M, Prakash Sharma P, Rathi B, Sharma S (2022). Mycobacterium tuberculosis dormancy regulon proteins Rv2627c and Rv2628 as Toll-like receptor agonist and as potential adjuvant. Int Immunopharmacol..

[91] Saelee C (2022). Toll-like receptor-mediated innate immune responses by recognition of the recombinant dormancy-associated Mycobacterium tuberculosis proteins Rv2659c and Rv1738. PLoS One.

[92] Mukamolova GV, Kaprelyants AS, Young DI, Young M, Kell DB (1998). A bacterial cytokine. Proc. Natl Acad. Sci. USA.

[93] Mukamolova GV (2002). The rpf gene of Micrococcus luteus encodes an essential secreted growth factor. Mol. Microbiol..

[94] Mukamolova GV (2002). A family of autocrine growth factors in Mycobacterium tuberculosis. Mol. Microbiol..

[95] Yeremeev VV (2003). Proteins of the Rpf family: immune cell reactivity and vaccination efficacy against tuberculosis in mice. Infect. Immun..

[96] Romano M (2012). Potential of Mycobacterium tuberculosis resuscitation-promoting factors as antigens in novel tuberculosis sub-unit vaccines. Microbes Infect..

[97] Lee J, Kim J, Lee J, Shin SJ, Shin EC (2014). DNA immunization of Mycobacterium tuberculosis resuscitation-promoting factor B elicits polyfunctional CD8(+) T cell responses. Clin. Exp. Vaccin. Res..

[98] Xin Q (2013). Subunit vaccine consisting of multi-stage antigens has high protective efficacy against Mycobacterium tuberculosis infection in mice. PLoS One.

[99] Ma J (2016). Mycobacterium tuberculosis multistage antigens confer comprehensive protection against pre- and post-exposure infections by driving Th1-type T cell immunity. Oncotarget.

[100] Yu Q, Wang X, Fan X (2017). A New Adjuvant MTOM Mediates Mycobacterium tuberculosis Subunit Vaccine to Enhance Th1-Type T Cell Immune Responses and IL-2(+) T Cells. Front. Immunol..

[101] Masungi C (2002). Differential T and B cell responses against Mycobacterium tuberculosis heparin-binding hemagglutinin adhesin in infected healthy individuals and patients with tuberculosis. J. Infect. Dis..

[102] Hougardy JM (2007). Heparin-binding-hemagglutinin-induced IFN-gamma release as a diagnostic tool for latent tuberculosis. PLoS One.

[103] Pethe K (2001). The heparin-binding haemagglutinin of M. tuberculosis is required for extrapulmonary dissemination. Nature.

[104] Menozzi FD (1996). Identification of a heparin-binding hemagglutinin present in mycobacteria. J. Exp. Med..

[105] Parra M (2004). The mycobacterial heparin-binding hemagglutinin is a protective antigen in the mouse aerosol challenge model of tuberculosis. Infect. Immun..

[106] Locht C, Hougardy JM, Rouanet C, Place S, Mascart F (2006). Heparin-binding hemagglutinin, from an extrapulmonary dissemination factor to a powerful diagnostic and protective antigen against tuberculosis. Tuberculosis.

[107] Rouanet C, Debrie AS, Lecher S, Locht C (2009). Subcutaneous boosting with heparin binding haemagglutinin increases BCG-induced protection against tuberculosis. Microbes Infect..

[108] Guerrero GG, Debrie AS, Locht C (2010). Boosting with mycobacterial heparin-binding haemagglutinin enhances protection of Mycobacterium bovis BCG-vaccinated newborn mice against M. tuberculosis. Vaccine.

[109] Guerrero GG, Locht C (2011). Recombinant HBHA boosting effect on BCG-induced immunity against Mycobacterium tuberculosis infection. Clin. Dev. Immunol..

[110] Young RA (1985). Dissection of Mycobacterium tuberculosis antigens using recombinant DNA. Proc. Natl Acad. Sci. USA.

[111] Results of a World Health Organization-sponsored workshop to characterize antigens recognized by mycobacterium-specific monoclonal antibodies. *Infect. Immun.* **51**, 718–720, (1986). 10.1128/iai.51.2.718-720.1986 PMC262422 3943911

[112] Young DB, Kent L, Young RA (1987). Screening of a recombinant mycobacterial DNA library with polyclonal antiserum and molecular weight analysis of expressed antigens. Infect. Immun..

[113] Vismara D (1990). Identification of a 35-kilodalton Mycobacterium tuberculosis protein containing B- and T-cell epitopes. Infect. Immun..

[114] Schou C, Yuan ZL, Andersen AB, Bennedsen J (1985). Production and partial characterization of monoclonal hybridoma antibodies to Mycobacterium tuberculosis. Acta Pathol. Microbiol Immunol. Scand. C..

[115] Andersen AB, Worsaae A, Chaparas SD (1988). Isolation and characterization of recombinant lambda gt11 bacteriophages expressing eight different mycobacterial antigens of potential immunological relevance. Infect. Immun..

[116] Kadival GV, Chaparas SD (1987). Production, characterization, and species specificity of five monoclonal antibodies to Mycobacterium tuberculosis. J. Clin. Microbiol..

[117] Ljungqvist L, Worsaae A, Heron I (1988). Antibody responses against Mycobacterium tuberculosis in 11 strains of inbred mice: novel monoclonal antibody specificities generated by fusions, using spleens from BALB.B10 and CBA/J mice. Infect. Immun..

[118] Worsaae A, Ljungqvist L, Heron I (1988). Monoclonal antibodies produced in BALB.B10 mice define new antigenic determinants in culture filtrate preparations of Mycobacterium tuberculosis. J. Clin. Microbiol..

[119] Worsaae A, Ljungqvist L, Haslov K, Heron I, Bennedsen J (1987). Allergenic and blastogenic reactivity of three antigens from Mycobacterium tuberculosis in sensitized guinea pigs. Infect. Immun..

[120] Skjot RL (2000). Comparative evaluation of low-molecular-mass proteins from Mycobacterium tuberculosis identifies members of the ESAT-6 family as immunodominant T-cell antigens. Infect. Immun..

[121] Sun R (2009). Novel recombinant BCG expressing perfringolysin O and the over-expression of key immunodominant antigens; pre-clinical characterization, safety and protection against challenge with Mycobacterium tuberculosis. Vaccine.

[122] Hess J (1998). Mycobacterium bovis Bacille Calmette-Guerin strains secreting listeriolysin of Listeria monocytogenes. Proc. Natl Acad. Sci. USA.

[123] Yang JD (2018). Mycobacterium tuberculosis-specific CD4+ and CD8+ T cells differ in their capacity to recognize infected macrophages. PLoS Pathog..

[124] Sutiwisesak R (2020). A natural polymorphism of Mycobacterium tuberculosis in the esxH gene disrupts immunodomination by the TB10.4-specific CD8 T cell response. PLoS Pathog..

[125] Skeiky YA (1999). Cloning, expression, and immunological evaluation of two putative secreted serine protease antigens of Mycobacterium tuberculosis. Infect. Immun..

[126] Tait DR (2019). Final Analysis of a Trial of M72/AS01(E) Vaccine to Prevent Tuberculosis. N. Engl. J. Med..

[127] Dillon DC (1999). Molecular characterization and human T-cell responses to a member of a novel Mycobacterium tuberculosis mtb39 gene family. Infect. Immun..

[128] Skeiky YA (2000). T cell expression cloning of a Mycobacterium tuberculosis gene encoding a protective antigen associated with the early control of infection. J. Immunol..

[129] Alderson MR (2000). Expression cloning of an immunodominant family of Mycobacterium tuberculosis antigens using human CD4(+) T cells. J. Exp. Med..

[130] Bavaro, T. et al. Glycosylation of recombinant antigenic proteins from mycobacterium tuberculosis: in silico prediction of protein epitopes and ex vivo biological evaluation of new semi-synthetic glycoconjugates. *Molecules* **22**, 10.3390/molecules22071081 (2017). 10.3390/molecules22071081 PMC6152100 28661444

[131] De Groot AS (2020). Better Epitope discovery, precision immune engineering, and accelerated vaccine design using immunoinformatics tools. Front Immunol..

[132] Cole ST (1998). Deciphering the biology of Mycobacterium tuberculosis from the complete genome sequence. Nature.

[133] Zvi A, Ariel N, Fulkerson J, Sadoff JC, Shafferman A (2008). Whole genome identification of Mycobacterium tuberculosis vaccine candidates by comprehensive data mining and bioinformatic analyses. BMC Med Genomics.

[134] Shafferman, A. et al. Recombinant BCG tuberculosis vaccine for eliciting immune responses to mycobacterium tuberculosis. Wo patent WO 2009/070700 A1 (2009).

[135] Lindestam Arlehamn CS (2013). Memory T cells in latent Mycobacterium tuberculosis infection are directed against three antigenic islands and largely contained in a CXCR3+CCR6+ Th1 subset. PLoS Pathog..

[136] Carpenter C (2015). A side-by-side comparison of T cell reactivity to fifty-nine Mycobacterium tuberculosis antigens in diverse populations from five continents. Tuberculosis.

[137] De Groot AS (2005). Developing an epitope-driven tuberculosis (TB) vaccine. Vaccine.

[138] Sweeney KA (2011). A recombinant Mycobacterium smegmatis induces potent bactericidal immunity against Mycobacterium tuberculosis. Nat. Med..

[139] Johnson, A. J. et al. Identification of Mycobacterial RplJ/L10 and RpsA/S1 Proteins as Novel Targets for CD4(+) T Cells. *Infect. Immun*. **85**, 10.1128/IAI.01023-16 (2017). 10.1128/IAI.01023-16 PMC5364311 28115505

[140] Kennedy, S. C. et al. Identification of mycobacterial ribosomal proteins as targets for CD4(+) T cells that enhance protective immunity in tuberculosis. *Infect Immun* **86**, 10.1128/IAI.00009-18 (2018). 10.1128/IAI.00009-18 PMC6105890 29891545

[141] Lin PL, Flynn JL (2015). CD8 T cells and Mycobacterium tuberculosis infection. Semin Immunopathol..

[142] Hammond AS (2005). Mycobacterium tuberculosis genome-wide screen exposes multiple CD8 T cell epitopes. Clin. Exp. Immunol..

[143] Tang ST (2011). Genome-based in silico identification of new Mycobacterium tuberculosis antigens activating polyfunctional CD8+ T cells in human tuberculosis. J. Immunol..

[144] Sundaramurthi JC (2012). In silico identification of potential antigenic proteins and promiscuous CTL epitopes in Mycobacterium tuberculosis. Infect. Genet. Evol..

[145] Lewinsohn DM (2013). Human Mycobacterium tuberculosis CD8 T Cell Antigens/Epitopes Identified by a Proteomic Peptide Library. PLoS One.

[146] Cohen GB (1999). The selective downregulation of class I major histocompatibility complex proteins by HIV-1 protects HIV-infected cells from NK cells. Immunity.

[147] Heinzel AS (2002). HLA-E-dependent presentation of Mtb-derived antigen to human CD8+ T cells. J. Exp. Med.

[148] Joosten SA (2010). Mycobacterium tuberculosis peptides presented by HLA-E molecules are targets for human CD8 T-cells with cytotoxic as well as regulatory activity. PLoS Pathog..

[149] van Meijgaarden KE (2015). Human CD8+ T-cells recognizing peptides from Mycobacterium tuberculosis (Mtb) presented by HLA-E have an unorthodox Th2-like, multifunctional, Mtb inhibitory phenotype and represent a novel human T-cell subset. PLoS Pathog..

[150] Caccamo N (2015). Human CD8 T lymphocytes recognize Mycobacterium tuberculosis antigens presented by HLA-E during active tuberculosis and express type 2 cytokines. Eur. J. Immunol..

[151] Commandeur S (2013). An unbiased genome-wide Mycobacterium tuberculosis gene expression approach to discover antigens targeted by human T cells expressed during pulmonary infection. J. Immunol..

[152] Commandeur S (2014). The in vivo expressed Mycobacterium tuberculosis (IVE-TB) antigen Rv2034 induces CD4(+) T-cells that protect against pulmonary infection in HLA-DR transgenic mice and guinea pigs. Vaccine.

[153] Coppola M (2016). New genome-wide algorithm identifies novel in-vivo expressed mycobacterium tuberculosis antigens inducing human T-cell responses with classical and unconventional cytokine profiles. Sci. Rep..

[154] Coppola M (2021). In-vivo expressed Mycobacterium tuberculosis antigens recognised in three mouse strains after infection and BCG vaccination. NPJ Vaccines.

[155] Lewinsohn DA (2007). Immunodominant tuberculosis CD8 antigens preferentially restricted by HLA-B. PLoS Pathog..

[156] Lewinsohn, D. A. et al. Comprehensive definition of human immunodominant CD8 antigens in tuberculosis. *NPJ Vaccines* **2**, 10.1038/s41541-017-0008-6 (2017). 10.1038/s41541-017-0008-6 PMC5538316 28775896

[157] Stylianou, E. et al. Identification and evaluation of novel protective antigens for the development of a candidate tuberculosis subunit vaccine. *Infect Immun* **86**, 10.1128/IAI.00014-18 (2018). 10.1128/IAI.00014-18 PMC6013653 29661928

[158] Huang H, Wang C, Rubelt F, Scriba TJ, Davis MM (2020). Analyzing the Mycobacterium tuberculosis immune response by T-cell receptor clustering with GLIPH2 and genome-wide antigen screening. Nat. Biotechnol..

[159] Musvosvi M (2023). T cell receptor repertoires associated with control and disease progression following Mycobacterium tuberculosis infection. Nat. Med.

[160] Purcell AW, Ramarathinam SH, Ternette N (2019). Mass spectrometry-based identification of MHC-bound peptides for immunopeptidomics. Nat. Protoc..

[161] Bettencourt P (2020). Identification of antigens presented by MHC for vaccines against tuberculosis. NPJ Vaccines.

[162] McMurtrey C (2017). T cell recognition of Mycobacterium tuberculosis peptides presented by HLA-E derived from infected human cells. PLoS One.

[163] Larrouy-Maumus G (2017). Protective efficacy of a lipid antigen vaccine in a guinea pig model of tuberculosis. Vaccine.

[164] Morgun, E. et al. Vaccination with mycobacterial lipid loaded nanoparticle leads to lipid antigen persistence and memory differentiation of antigen-specific T cells. *bioRxiv*, 10.1101/2023.03.07.531489 (2023). 10.7554/eLife.87431 PMC10599656 37877801

[165] Mehaffy C, Ryan JM, Kruh-Garcia NA, Dobos KM (2022). Extracellular Vesicles in Mycobacteria and Tuberculosis. Front Cell Infect. Microbiol.

[166] Prados-Rosales R (2014). Mycobacterial membrane vesicles administered systemically in mice induce a protective immune response to surface compartments of Mycobacterium tuberculosis. mBio.

[167] Tirado Y (2016). Mycobacterium smegmatis proteoliposome induce protection in a murine progressive pulmonary tuberculosis model. Tuberculosis.

[168] George E (2022). Immunomodulatory effect of mycobacterial outer membrane vesicles coated nanoparticles. Biomater. Adv..

[169] Phuah JY, Mattila JT, Lin PL, Flynn JL (2012). Activated B cells in the granulomas of nonhuman primates infected with Mycobacterium tuberculosis. Am. J. Pathol..

[170] Alvarez N (2013). Passive administration of purified secretory IgA from human colostrum induces protection against Mycobacterium tuberculosis in a murine model of progressive pulmonary infection. BMC Immunol..

[171] Balu S (2011). A novel human IgA monoclonal antibody protects against tuberculosis. J. Immunol..

[172] Kumar SK, Singh P, Sinha S (2015). Naturally produced opsonizing antibodies restrict the survival of Mycobacterium tuberculosis in human macrophages by augmenting phagosome maturation. Open Biol..

[173] Li H (2017). Latently and uninfected healthcare workers exposed to TB make protective antibodies against Mycobacterium tuberculosis. Proc. Natl Acad. Sci. USA.

[174] Lopez Y (2009). Induction of a protective response with an IgA monoclonal antibody against Mycobacterium tuberculosis 16kDa protein in a model of progressive pulmonary infection. Int J. Med Microbiol.

[175] Lu LL (2016). A functional role for antibodies in tuberculosis. Cell.

[176] Lu LL (2019). IFN-gamma-independent immune markers of Mycobacterium tuberculosis exposure. Nat. Med..

[177] Williams A (2004). Passive protection with immunoglobulin A antibodies against tuberculous early infection of the lungs. Immunology.

[178] Zimmermann N (2016). Human isotype-dependent inhibitory antibody responses against Mycobacterium tuberculosis. EMBO Mol. Med.

[179] Brown RM (2003). Lipoarabinomannan-reactive human secretory immunoglobulin A responses induced by mucosal bacille Calmette-Guerin vaccination. J. Infect. Dis..

[180] Li H, Javid B (2018). Antibodies and tuberculosis: finally coming of age?. Nat. Rev. Immunol..

[181] Goodswen, S. J., Kennedy, P. J. & Ellis, J. T. A guide to current methodology and usage of reverse vaccinology towards in silico vaccine discovery. *FEMS Microbiol Rev* **47**, 10.1093/femsre/fuad004 (2023). 10.1093/femsre/fuad004 36806618

[182] Li W, Joshi MD, Singhania S, Ramsey KH, Murthy AK (2014). Peptide vaccine: progress and challenges. Vaccines.

[183] Bibi S (2021). In silico analysis of epitope-based vaccine candidate against tuberculosis using reverse vaccinology. Sci. Rep..

[184] Khan Z (2022). Insight Into novel anti-tuberculosis vaccines by using immunoinformatics approaches. Front. Microbiol..

[185] Peng, C. et al. Immunoinformatic-based multi-epitope vaccine design for co-infection of mycobacterium tuberculosis and SARS-CoV-2. *J. Pers. Med.* **13**, 10.3390/jpm13010116 (2023). 10.3390/jpm13010116 PMC9863242 36675777

[186] Sharma R, Rajput VS, Jamal S, Grover A, Grover S (2021). An immunoinformatics approach to design a multi-epitope vaccine against Mycobacterium tuberculosis exploiting secreted exosome proteins. Sci. Rep..

[187] Cheng P (2023). Bioinformatics analysis and consistency verification of a novel tuberculosis vaccine candidate HP13138PB. Front. Immunol..

[188] Jiang, F. et al. PP19128R, a multiepitope vaccine designed to prevent latent tuberculosis infection, induced immune responses in silico and in vitro assays. *Vaccines* **11**, 10.3390/vaccines11040856 (2023). 10.3390/vaccines11040856 PMC10145841 37112768

[189] Al Tbeishat H (2022). Novel In Silico mRNA vaccine design exploiting proteins of M. tuberculosis that modulates host immune responses by inducing epigenetic modifications. Sci. Rep..

[190] Larsen, S. E. et al. An RNA-Based Vaccine Platform for Use against Mycobacterium tuberculosis. *Vaccines* **11**, 10.3390/vaccines11010130 (2023). 10.3390/vaccines11010130 PMC9862644 36679975

[191] Bucsan, A. N., Mehra, S., Khader, S. A. & Kaushal, D. The current state of animal models and genomic approaches towards identifying and validating molecular determinants of Mycobacterium tuberculosis infection and tuberculosis disease. *Pathog. Dis.* **77**, 10.1093/femspd/ftz037 (2019). 10.1093/femspd/ftz037 PMC6687098 31381766

[192] Gong W, Liang Y, Wu X (2020). Animal models of tuberculosis vaccine research: an important component in the fight against tuberculosis. Biomed. Res. Int..

[193] Singh AK, Gupta UD (2018). Animal models of tuberculosis: Lesson learnt. Indian J. Med. Res.

[194] Tameris M (2014). The candidate TB vaccine, MVA85A, induces highly durable Th1 responses. PLoS One.

[195] Dye C, Fine PE (2013). A major event for new tuberculosis vaccines. Lancet.

[196] Macleod M (2018). Learning lessons from MVA85A, a failed booster vaccine for BCG. BMJ.

[197] Andersen P, Doherty TM (2005). The success and failure of BCG - implications for a novel tuberculosis vaccine. Nat. Rev. Microbiol..

[198] Irvine EB (2021). Robust IgM responses following intravenous vaccination with Bacille Calmette-Guerin associate with prevention of Mycobacterium tuberculosis infection in macaques. Nat. Immunol..

[199] Larson EC (2023). Intravenous Bacille Calmette-Guerin vaccination protects simian immunodeficiency virus-infected macaques from tuberculosis. Nat. Microbiol..

[200] Marwick C (1998). Volunteers in typhoid infection study will aid future vaccine development. JAMA.

[201] Carrat F (2008). Time lines of infection and disease in human influenza: a review of volunteer challenge studies. Am. J. Epidemiol..

[202] Statler J, Mammen M, Lyons A, Sun W (2008). Sonographic findings of healthy volunteers infected with dengue virus. J. Clin. Ultrasound.

[203] Sauerwein RW, Roestenberg M, Moorthy VS (2011). Experimental human challenge infections can accelerate clinical malaria vaccine development. Nat. Rev. Immunol..

[204] Garnier T (2003). The complete genome sequence of Mycobacterium bovis. Proc. Natl Acad. Sci. USA.

[205] Kleinwaks G, Schmit V, Morrison J (2022). Considering human challenge trials for tuberculosis vaccine development. Vaccine.

[206] Chan CT, Lee JW, Cameron DE, Bashor CJ, Collins JJ (2016). Deadman’ and ‘Passcode’ microbial kill switches for bacterial containment. Nat. Chem. Biol..

[207] Callura JM, Dwyer DJ, Isaacs FJ, Cantor CR, Collins JJ (2010). Tracking, tuning, and terminating microbial physiology using synthetic riboregulators. Proc. Natl Acad. Sci. USA.

[208] Contreras A, Molin S, Ramos JL (1991). Conditional-suicide containment system for bacteria which mineralize aromatics. Appl Environ. Microbiol.

[209] Minassian AM (2012). A human challenge model for Mycobacterium tuberculosis using Mycobacterium bovis bacille Calmette-Guerin. J. Infect. Dis..

[210] Chen L, Wang J, Zganiacz A, Xing Z (2004). Single intranasal mucosal Mycobacterium bovis BCG vaccination confers improved protection compared to subcutaneous vaccination against pulmonary tuberculosis. Infect. Immun..

[211] Lagranderie MR, Balazuc AM, Deriaud E, Leclerc CD, Gheorghiu M (1996). Comparison of immune responses of mice immunized with five different Mycobacterium bovis BCG vaccine strains. Infect. Immun..

[212] Dobakhti F (2009). Adjuvanticity effect of sodium alginate on subcutaneously injected BCG in BALB/c mice. Microbes Infect..

[213] Goonetilleke NP (2003). Enhanced immunogenicity and protective efficacy against Mycobacterium tuberculosis of bacille Calmette-Guerin vaccine using mucosal administration and boosting with a recombinant modified vaccinia virus Ankara. J. Immunol..

[214] Williams A (2005). Evaluation of vaccines in the EU TB Vaccine Cluster using a guinea pig aerosol infection model of tuberculosis. Tuberculosis (Edinb.).

[215] Harris SA (2014). Evaluation of a human BCG challenge model to assess antimycobacterial immunity induced by BCG and a candidate tuberculosis vaccine, MVA85A, alone and in combination. J. Infect. Dis..

[216] McShane H (2004). Recombinant modified vaccinia virus Ankara expressing antigen 85A boosts BCG-primed and naturally acquired antimycobacterial immunity in humans. Nat. Med..

[217] Davids M (2020). A Human Lung Challenge Model to Evaluate the Safety and Immunogenicity of PPD and Live Bacillus Calmette-Guerin. Am. J. Respir. Crit. Care Med.

[218] Wang, X. et al. Development of an engineered mycobacterium tuberculosis strain for a safe and effective tuberculosis human challenge model. *bioRxiv*, 10.1101/2023.11.19.567569 (2023).

[219] Shamir ER, Ewald AJ (2014). Three-dimensional organotypic culture: experimental models of mammalian biology and disease. Nat. Rev. Mol. Cell Biol..

[220] Silva Miranda M, Breiman A, Allain S, Deknuydt F, Altare F (2012). The tuberculous granuloma: an unsuccessful host defence mechanism providing a safety shelter for the bacteria?. Clin. Dev. Immunol..

[221] Puissegur MP (2004). An in vitro dual model of mycobacterial granulomas to investigate the molecular interactions between mycobacteria and human host cells. Cell Microbiol.

[222] Husain AA, Rajpal S (2012). Evaluation and identification of in vitro cellular immune response to culture filtrate antigens of M. tuberculosis culture. Implic. Vaccin. Des. J. Vaccines Vaccin.

[223] Kapoor N (2013). Human granuloma in vitro model, for TB dormancy and resuscitation. PLoS One.

[224] Nguyen Hoang AT (2012). Dendritic cell functional properties in a three-dimensional tissue model of human lung mucosa. Am. J. Physiol. Lung Cell Mol. Physiol..

[225] Parasa VR (2014). Modeling Mycobacterium tuberculosis early granuloma formation in experimental human lung tissue. Dis. Model Mech..

[226] Braian, C., Svensson, M., Brighenti, S., Lerm, M. & Parasa, V. R. A 3D human lung tissue model for functional studies on mycobacterium tuberculosis infection. *J Vis Exp*, 10.3791/53084 (2015). 10.3791/53084 PMC4692636 26485646

[227] Abel L, El-Baghdadi J, Bousfiha AA, Casanova JL, Schurr E (2014). Human genetics of tuberculosis: a long and winding road. Philos. Trans. R. Soc. Lond. B Biol. Sci..

[228] Medina E, North RJ (1998). Resistance ranking of some common inbred mouse strains to Mycobacterium tuberculosis and relationship to major histocompatibility complex haplotype and Nramp1 genotype. Immunology.

[229] Kramnik I, Dietrich WF, Demant P, Bloom BR (2000). Genetic control of resistance to experimental infection with virulent Mycobacterium tuberculosis. Proc. Natl Acad. Sci. USA.

[230] Pan H (2005). Ipr1 gene mediates innate immunity to tuberculosis. Nature.

[231] North RJ, Medina E (1998). How important is Nramp1 in tuberculosis?. Trends Microbiol..

[232] Malo D (1994). Haplotype mapping and sequence analysis of the mouse Nramp gene predict susceptibility to infection with intracellular parasites. Genomics.

[233] Vidal SM, Malo D, Vogan K, Skamene E, Gros P (1993). Natural resistance to infection with intracellular parasites: isolation of a candidate for Bcg. Cell.

[234] Fortin A, Abel L, Casanova JL, Gros P (2007). Host genetics of mycobacterial diseases in mice and men: forward genetic studies of BCG-osis and tuberculosis. Annu Rev. Genomics Hum. Genet..

[235] Apt A, Kramnik I (2009). Man and mouse TB: contradictions and solutions. Tuberculosis.

[236] Svenson KL (2012). High-resolution genetic mapping using the Mouse Diversity outbred population. Genetics.

[237] Niazi MK (2015). Lung necrosis and neutrophils reflect common pathways of susceptibility to Mycobacterium tuberculosis in genetically diverse, immune-competent mice. Dis. Model Mech..

[238] Gopal R (2013). S100A8/A9 proteins mediate neutrophilic inflammation and lung pathology during tuberculosis. Am. J. Respir. Crit. Care Med.

[239] Ahmed, M. et al. Immune correlates of tuberculosis disease and risk translate across species. *Sci. Transl. Med.* **12**, 10.1126/scitranslmed.aay0233 (2020). 10.1126/scitranslmed.aay0233 PMC7354419 31996462

[240] Kurtz, S. L. et al. The diversity outbred mouse population is an improved animal model of vaccination against tuberculosis that reflects heterogeneity of protection. *mSphere* **5**, 10.1128/mSphere.00097-20 (2020). 10.1128/mSphere.00097-20 PMC7160682 32295871

[241] Kurtz SL (2023). Intravenous BCG vaccination of diversity outbred mice results in moderately enhanced protection against challenge with Mycobacterium tuberculosis compared to intradermal vaccination. Infect. Immun..

[242] Lai, R. et al. Host genetic background is a barrier to broadly effective vaccine-mediated protection against tuberculosis. *J. Clin. Invest.* **133**, 10.1172/JCI167762 (2023). 10.1172/JCI167762 PMC10313364 37200108

[243] Smith, C. M. et al. Host-pathogen genetic interactions underlie tuberculosis susceptibility in genetically diverse mice. *Elife* **11**, 10.7554/eLife.74419 (2022). 10.7554/eLife.74419 PMC8846590 35112666

[244] Saul MC, Philip VM, Reinholdt LG, Chesler EJ, Center for Systems Neurogenetics of, A (2019). High-diversity mouse populations for complex traits. Trends Genet.

[245] Threadgill DW, Miller DR, Churchill GA, de Villena FP (2011). The collaborative cross: a recombinant inbred mouse population for the systems genetic era. ILAR J..

[246] Churchill GA (2004). The Collaborative Cross, a community resource for the genetic analysis of complex traits. Nat. Genet..

[247] Churchill GA, Gatti DM, Munger SC, Svenson KL (2012). The diversity outbred mouse population. Mamm. Genome.

[248] Plumlee CR (2021). Ultra-low dose aerosol infection of mice with mycobacterium tuberculosis more closely models human tuberculosis. Cell Host Microbe.

[249] Balasubramanian V, Wiegeshaus EH, Taylor BT, Smith DW (1994). Pathogenesis of tuberculosis: pathway to apical localization. Tube. Lung Dis..

[250] Donald PR (2018). Droplets, dust and guinea pigs: an historical review of tuberculosis transmission research, 1878-1940. Int J. Tuberc. Lung Dis..

[251] Vidal SJ (2023). Attenuated Mycobacterium tuberculosis vaccine protection in a low-dose murine challenge model. iScience.

[252] Bosma GC, Custer RP, Bosma MJ (1983). A severe combined immunodeficiency mutation in the mouse. Nature.

[253] Heuts F (2013). CD4+ cell-dependent granuloma formation in humanized mice infected with mycobacteria. Proc. Natl Acad. Sci. USA.

[254] Calderon VE (2013). A humanized mouse model of tuberculosis. PLoS One.

[255] Lee J, Brehm MA, Greiner D, Shultz LD, Kornfeld H (2013). Engrafted human cells generate adaptive immune responses to Mycobacterium bovis BCG infection in humanized mice. BMC Immunol..

[256] Grover A (2017). Humanized NOG mice as a model for tuberculosis vaccine-induced immunity: a comparative analysis with the mouse and guinea pig models of tuberculosis. Immunology.

[257] Lepore M, Mori L, De Libero G (2018). The conventional nature of non-MHC-restricted T cells. Front Immunol..

[258] Zhao, J. et al. Mycolic acid-specific T cells protect against Mycobacterium tuberculosis infection in a humanized transgenic mouse model. *Elife* **4**, 10.7554/eLife.08525 (2015). 10.7554/eLife.08525 PMC4718816 26652001

[259] Smaill F (2013). A human type 5 adenovirus-based tuberculosis vaccine induces robust T cell responses in humans despite preexisting anti-adenovirus immunity. Sci. Transl. Med..

[260] Yao Y (2017). Enhancement of antituberculosis immunity in a humanized model system by a novel virus-vectored respiratory mucosal vaccine. J. Infect. Dis..

[261] Myllymaki H (2017). Identification of novel antigen candidates for a tuberculosis vaccine in the adult zebrafish (Danio rerio). PLoS One.

[262] Tobin DM (2010). The lta4h locus modulates susceptibility to mycobacterial infection in zebrafish and humans. Cell.

[263] Oksanen KE (2013). An adult zebrafish model for preclinical tuberculosis vaccine development. Vaccine.

[264] Oksanen KE (2016). DNA vaccination boosts Bacillus Calmette-Guerin protection against mycobacterial infection in zebrafish. Dev. Comp. Immunol..

[265] Cui Z, Samuel-Shaker D, Watral V, Kent ML (2010). Attenuated Mycobacterium marinum protects zebrafish against mycobacteriosis. J. Fish. Dis..

[266] Ramakrishnan L, Federspiel NA, Falkow S (2000). Granuloma-specific expression of Mycobacterium virulence proteins from the glycine-rich PE-PGRS family. Science.

[267] Myllymaki, H., Niskanen, M., Luukinen, H., Parikka, M. & Ramet, M. Identification of protective postexposure mycobacterial vaccine antigens using an immunosuppression-based reactivation model in the zebrafish. *Dis Model Mech.* **11**, 10.1242/dmm.033175 (2018). 10.1242/dmm.033175 PMC5897733 29590635

[268] Meijer AH, Spaink HP (2011). Host-pathogen interactions made transparent with the zebrafish model. Curr. Drug Targets.

[269] Dockrell HM, Smith SG (2017). What Have We Learnt about BCG Vaccination in the Last 20 Years?. Front Immunol..

[270] Geluk A (2007). T-cell recognition of the HspX protein of Mycobacterium tuberculosis correlates with latent M. tuberculosis infection but not with M. bovis BCG vaccination. Infect. Immun..

[271] Lin MY (2007). Lack of immune responses to Mycobacterium tuberculosis DosR regulon proteins following Mycobacterium bovis BCG vaccination. Infect. Immun..

[272] Ottenhoff THM, Joosten SA (2019). Mobilizing unconventional T cells. Science.

